# Selpercatinib mitigates microglia-mediated neuroinflammation by inhibiting RET signaling and NLRP3 inflammasome activation

**DOI:** 10.3389/fimmu.2026.1899666

**Published:** 2026-09-23

**Authors:** Jung Ki Min, Daeun Park, Hanwoong Woo, Sohee Kim, Juyeon Lee, Yeonsu Kim, Jeong-im Choi, Eunbi Lim, Sehyun Chae, Jieun Kim

**Affiliations:** 1 Multidimensional Genomics Research Center, Kangwon National University, Chuncheon, Republic of Korea; 2 Department of Bio-Health Convergence, Kangwon National University, Chuncheon, Republic of Korea; 3 Division of Chemical Engineering and Bioengineering, College of Art Culture and Engineering, Kangwon National University, Chuncheon, Republic of Korea; 4 Department of Bio-Health Technology, College of Biomedical Science, Kangwon National University, Chuncheon, Republic of Korea

**Keywords:** LPS, microglia, neuroinflammation, NLRP3 inflammasome, RET, Selpercatinib

## Abstract

**Background:**

Selpercatinib is a highly selective rearranged during transfection (RET) receptor tyrosine kinase inhibitor approved for RET fusion-positive cancers and known to penetrate the blood–brain barrier. Although RET signaling has been implicated in neural development and cell survival, its role in microglia-mediated neuroinflammation and NOD-like receptor family pyrin domain-containing 3 (NLRP3) inflammasome activation remains poorly understood. This study investigated whether Selpercatinib attenuates neuroinflammatory signaling in microglia by modulating RET-dependent kinase cascades.

**Methods:**

Neuroinflammation was induced in BV2 microglial cells using lipopolysaccharide (LPS; 200 ng/mL), followed by post-treatment with Selpercatinib (5 μM). For *in vivo* validation, male C57BL/6N mice received systemic LPS administration (10 mg/kg, i.p.) followed by Selpercatinib treatment (10 mg/kg, i.p.), and hippocampal tissues were collected for analysis. Proinflammatory gene expression, RET-associated signaling pathways, microglial phenotypic markers, and NLRP3 inflammasome components were assessed using quantitative real-time polymerase chain reaction, western blotting, enzyme-linked immunosorbent assay, immunocytochemistry, and flow cytometry. RET-targeting siRNA was used to genetically validate the involvement of RET in LPS-induced inflammatory signaling.

**Results:**

Selpercatinib reduced RET phosphorylation at Tyr1062 in LPS-stimulated BV2 microglia and reduced the mRNA expression of proinflammatory mediators, including *il-1β, il-6, tnf-α, cox-2*, *inos, ccl-2*, and *ccl-7* in BV2 microglia. Mechanistically, Selpercatinib attenuated AKT and ERK phosphorylation, inhibited NF-κB nuclear translocation, and reduced CREB phosphorylation. Similarly, RET knockdown reduced LPS-induced proinflammatory gene expression and attenuated AKT/ERK–NF-κB/CREB signaling, providing genetic evidence for the involvement of RET in these inflammatory responses. Selpercatinib also modulated LPS-induced microglial activation by decreasing the expression of reactive microglial markers, including CD16/32, disease-associated microglia-associated Clec7a, and microglial neurodegenerative phenotype-associated Galectin-3, while increasing CD206, the marker associated with tissue repair. In addition, Selpercatinib significantly reduced LPS-induced intracellular ROS generation. Furthermore, Selpercatinib inhibited NLRP3 inflammasome activation, as evidenced by reduced levels of NLRP3, ASC, pro-caspase-1, cleaved caspase-1, GSDMD, and IL-1β in BV2 microglia. Consistent with the *in vitro* findings, Selpercatinib attenuated LPS-induced hippocampal *il-1β* gene expression *in vivo* and reduced NLRP3 inflammasome-related proteins, including ASC, pro-caspase-1, cleaved caspase-1 and GSDMD-F and N in the hippocampus of mice.

**Conclusion:**

Selpercatinib attenuated the development of microglia-mediated neuroinflammatory responses through modulation of RET-associated AKT/ERK–NF-κB/CREB signaling and suppression of NLRP3 inflammasome activation. These findings provide a mechanistic basis for further investigating Selpercatinib as an early intervention agent for neuroinflammatory responses.

## 1 Introduction

Selpercatinib is a highly selective rearranged during transfection (RET) kinase inhibitor approved for RET fusion–positive malignancies, including non-small cell lung cancer and thyroid cancer (1, 2). RET is a receptor tyrosine kinase essential for neural development, cell survival, and differentiation through activation by glial cell line-derived neurotrophic factor (GDNF) family ligands (3, 4). Notably, Selpercatinib has been shown to penetrate the blood–brain barrier, supporting its potential to exert pharmacological effects within the central nervous system (CNS) (5, 6). Although RET signaling has been extensively characterized in oncogenesis, its contribution to neuroinflammatory signaling remains unclear. Determining whether RET inhibition modulates CNS inflammatory responses may therefore clarify an important neuropharmacological mechanism.

Neuroinflammation is a tightly regulated CNS process involving resident immune-cell activation and proinflammatory mediator production (7, 8). Microglia, the primary immune cells of the CNS, initiate and propagate neuroinflammatory responses after exposure to stimuli such as lipopolysaccharide (LPS) (9, 10). Activated microglia release cytokines including tumor necrosis factor (TNF-α), interleukin (IL-6), and IL-1β, as well as inducible nitric oxide synthase (iNOS) and cyclooxygenase-2 (COX-2), thereby amplifying inflammatory signaling cascades (11–13). These responses are mediated through intracellular pathways such as AKT, ERK, and nuclear factor-κB (NF-κB), which regulate inflammatory gene transcription (13, 14). Dysregulated neuroinflammation contributes to multiple neurological disorders, highlighting the need for therapeutic strategies that suppress excessive inflammatory activation.

Among key molecular drivers of neuroinflammation, inflammasomes have emerged as central regulators of innate immune responses in the CNS (15, 16). The NOD-like receptor family pyrin domain-containing 3 (NLRP3) inflammasome is a well-characterized inflammasome complex activated by various pathogen- and danger-associated signals (17, 18). After activation, NLRP3 recruits the adaptor protein apoptosis-associated speck-like protein containing a CARD (ASC), leading to pro-caspase-1 cleavage into its active form (17, 19, 20). Activated caspase-1 subsequently processes pro-IL-1β into its mature form and cleaves gasdermin D (GSDMD), thereby amplifying inflammatory responses (21–23). Despite the established role of inflammasomes in neuroinflammation, mechanisms linking RET receptor tyrosine kinase signaling to inflammasome activation remain unclear.

Microglia exhibit phenotypic diversity and functional plasticity in response to environmental and pathological cues (24). Transcriptomic profiling has identified multiple microglial states, including homeostatic microglia, disease-associated microglia (DAM), and neurodegenerative microglia (MGnD), with distinct molecular signatures and functions (25, 26). These states reflect context-dependent responses to injury, infection, and neurodegenerative stimuli within the CNS. Proinflammatory activation is often associated with increased expression of CD16/32, Clec7a, and Galectin-3, which are linked to DAM- and MGnD-like profiles and contribute to inflammatory signaling and phagocytic activity (27–29). Conversely, anti-inflammatory or tissue repair-associated microglial states are commonly characterized by CD206 expression, which is associated with inflammation resolution and restoration of tissue homeostasis (30). Clec7a and Galectin-3 are key markers of DAM and reflect a transition toward activated states in neurodegenerative conditions (28, 29, 31). Maintaining the balance of heterogeneous microglial states is essential for CNS homeostasis, whereas dysregulation toward proinflammatory or disease-associated phenotypes promotes neuroinflammation, supporting microglial state modulation as a therapeutic strategy (32).

Despite growing recognition of RET signaling in cellular regulation, its role in microglia-mediated neuroinflammation, inflammasome activation, and microglial functional responses remains unclear. This study examined the effects of Selpercatinib on LPS-induced neuroinflammatory responses in BV2 microglia and an acute neuroinflammation mouse model. Specifically, it assessed whether RET inhibition attenuates inflammatory signaling, suppresses NLRP3 inflammasome activation, and modulates microglial activation profiles. These findings offer new insight into the therapeutic potential of Selpercatinib for neuroinflammation-associated CNS disorders.

## 2 Materials and methods

### 2.1 Ethics statement

All animal experiments were conducted in accordance with the Institutional Animal Care and Use Committee (IACUC) guidelines of Kangwon National University (Chuncheon, Republic of Korea) and approved under protocol number KW-230907-3.

### 2.2 Selpercatinib and LPS treatments

Lipopolysaccharide (LPS; Escherichia coli O111, Sigma-Aldrich, St. Louis, MO, USA) was dissolved in phosphate-buffered saline (PBS) to induce inflammatory responses. For *in vitro* experiments, BV2 microglial cells were stimulated with LPS at 200 ng/mL. Selpercatinib (Selleck Chemicals, Cat. No. S8781, Houston, TX, USA) was dissolved in dimethyl sulfoxide (DMSO) and applied at a final concentration of 5 μM. For post-treatment conditions, cells were exposed to LPS and treated with Selpercatinib 30 min after LPS stimulation, unless otherwise specified.

For *in vivo* experiments, acute neuroinflammation was induced by intraperitoneal (i.p.) injection of LPS (10 mg/kg). Selpercatinib (1, 5, or 10 mg/kg, i.p.) or vehicle was administered 30 min after LPS injection. Mice received additional Selpercatinib or vehicle injections at 2.5- or 8-h intervals and were euthanized 2.5 or 8 h after the final administration. The hippocampus was rapidly isolated for subsequent analyses. For *in vivo* administration, Selpercatinib was prepared in a vehicle solution containing 10% DMSO, 40% polyethylene glycol 300 (PEG300), and 5% Tween 80 in deionized water.

### 2.3 BV2 microglial cells

BV2 microglial cells (RRID: CVCL_0182) were obtained from Cytion (Cat. No. 305156, Eppelheim, Germany) and maintained in high-glucose Dulbecco’s modified Eagle medium (DMEM; WELGENE, Cat. No. LM 001-07, Gyeongsan, Republic of Korea) supplemented with 5% fetal bovine serum (FBS; Gibco, Cat. No. 16000-044, Waltham, MA, USA), 100 U/mL penicillin G, and 100 μg/mL streptomycin. Cultures were kept at 37 °C in a humidified incubator with 5% CO_2_. Cells were routinely screened for mycoplasma contamination using the MycoStrip^®^ Mycoplasma Detection Kit (InvivoGen, Cat. No. rep-mys-20, San Diego, CA, USA) and confirmed to be mycoplasma-free.

### 2.4 Secreted GDNF concentration

To assess secreted glial cell line-derived neurotrophic factor (GDNF), conditioned medium was collected from BV2 microglial cells after LPS treatment for 45 min, 6 h, or 24 h. The collected medium was initially centrifuged at 2,300 rpm for 5 min at 4 °C, and the supernatant was transferred to a new tube and further centrifuged at 4,600 rpm for 10 min at 4 °C to remove residual cellular debris. The resulting supernatant was passed through a 0.22-μm filter and concentrated by centrifugation at 13,000 rpm for 5 min at 4 °C, using a centrifugal filter unit with a 10-kDa molecular weight cutoff (Cat. No. UFC5010, Millipore, Burlington, MA, USA). Equal volumes of the concentrated conditioned medium were mixed with 5× SDS sample buffer to achieve a final concentration of 1× SDS sample buffer, and the samples were heated at 100 °C for 10 min. Secreted GDNF levels were subsequently analyzed by western blotting.

### 2.5 RET siRNA transfection

To determine whether the effects of Selpercatinib on LPS-induced inflammatory responses are dependent on RET signaling, RET knockdown was performed in BV2 microglial cells using reverse transfection. To form siRNA–lipid complexes for transfection, RET-targeting siRNA (siRET; Cat. No. L-047013-00-0005, Dharmacon, Lafayette, CO, USA) or scrambled control siRNA (siSCR; Cat. No. D-001810-01-05, Dharmacon) was diluted in Opti-MEM medium (Cat. No. 31985-070, Gibco, Thermo Fisher Scientific, Waltham, MA, USA) and incubated with 1 μL of Lipofectamine^®^ RNAiMAX reagent (Cat. No. 13778-030, Invitrogen, Waltham, MA, USA) for 40 min at room temperature. The final siRNA concentration was 100 nM. The siRNA–lipid complexes were then added to 24-well culture plates, followed by seeding of BV2 microglial cells at 2 × 10^5^ cells per well. Depending on the experimental conditions, LPS and Selpercatinib treatments were initiated at the indicated time points so that all experimental endpoints were reached 48 h after siRNA transfection.

### 2.6 Cell counting kit-8 assay

BV2 cells were seeded in 96-well plates at 2 × 10^4^ cells per well and allowed to stabilize in serum-free medium for 1 h. Cells were then treated with Selpercatinib (0.001, 0.01, 0.1, 1, 2.5, 5, and 10 μM) or 1% DMSO vehicle for 24 h. To evaluate cell viability under the combined inflammatory treatment condition, BV2 microglia were co-treated with LPS (200 ng/mL) and Selpercatinib (5 μM) for 24 h. Cells treated with PBS, LPS alone, and Selpercatinib alone were included as corresponding controls. After treatment, CCK-8 (Dojindo, Cat. No. CK04, Kumamoto, Japan) solution was added to each well at 10% of the total volume, and plates were incubated for an additional 2 h. Cell viability was determined by measuring absorbance at 450 nm using a BioTek Epoch 2 microplate spectrophotometer (Agilent BioTek, Santa Clara, CA, USA).

### 2.7 Quantitative real-time polymerase chain reaction

Inflammation-related gene expression, including *il-1β, il-6, tnf-α, cox-2, inos, ccl-2*, and *ccl-7*, was analyzed in BV2 microglial cells and hippocampal tissues using quantitative real-time PCR (qPCR). Total RNA was isolated using TRIzol reagent (Invitrogen, Cat. No. 15596018, Waltham, MA, USA) according to the manufacturer’s protocol. First-strand cDNA was synthesized using SuPrimeScript RT Premix (GeNetBio, Cat. No. SR-5000, Daejeon, Republic of Korea). Quantitative PCR was performed with TOPreal™ SYBR Green qPCR PreMIX (Enzynomics, Cat. No. RT500M, Daejeon, Republic of Korea) on a QuantStudio™ 3 Real-Time PCR System (Thermo Fisher Scientific, Waltham, MA, USA) for 40 amplification cycles. Relative gene expression was calculated by the comparative Ct (2^−ΔΔCt^) method, using *gapdh* as the internal reference gene. Expression levels in LPS- or LPS + Selpercatinib-treated samples were normalized to those of vehicle-treated controls. Primer sequences are provided in **Supplementary Table 1**.

### 2.8 Cytosolic and nuclear fractionation

To assess phosphorylated NF-κB (p-NF-κB) nuclear translocation, BV2 microglial cells were exposed to LPS (200 ng/mL) or PBS for 30 min, followed by Selpercatinib (5 μM) or 1% DMSO vehicle treatment for 5.5 h. For subcellular fractionation, cells were collected, resuspended in cytosolic extraction buffer containing 10 mM HEPES (pH 7.4), 10 mM KCl, and 0.05% NP-40, and incubated on ice for 20 min. Samples were centrifuged at 13,000 rpm for 10 min at 4 °C to separate the cytosolic fraction. The supernatant was transferred to a new tube as the cytosolic fraction. The remaining pellet was resuspended in radioimmunoprecipitation assay (RIPA) buffer (Thermo Scientific, Cat. No. 89901, Waltham, MA, USA) and sonicated to disrupt nuclear membranes. After incubation on ice for 10 min, lysates were centrifuged at 13,000 rpm for 10 min at 4 °C. The supernatant was collected as the nuclear fraction and used for western blot analysis of p-NF-κB.

### 2.9 Western blotting

BV2 microglial cells were treated with Selpercatinib or 1% DMSO vehicle after stimulation with LPS or PBS, as described above. Cells were lysed in radioimmunoprecipitation assay (RIPA) buffer, briefly sonicated, and centrifuged at 13,000 rpm for 15 min at 4 °C to obtain clarified protein extracts. Protein concentrations were determined by BCA assay using bovine serum albumin as the standard. Equal protein amounts (30 μg per sample) were separated on 8–12% SDS-polyacrylamide gels and transferred onto polyvinylidene difluoride (PVDF) membranes (Immobilon^®^-P, MilliporeSigma, Burlington, MA, USA). Membranes were blocked with 5% skim milk for 1 h at room temperature and incubated overnight at 4 °C with primary antibodies against phospho-RET (Y1062), RET, phospho-AKT (S473), AKT, phospho-ERK1/2 (T202/Y204), ERK1/2, phospho-NF-κB (S536), NF-κB, phospho-CREB (S133), CREB, NLRP3, ASC, caspase-1, GSDMD, GDNF, GAPDH, and histone H3. After washing, membranes were incubated for 1 h at room temperature with horseradish peroxidase (HRP)-conjugated secondary antibodies, including goat anti-mouse IgG (Invitrogen, Carlsbad, CA, USA) or goat anti-rabbit IgG (Promega, Madison, WI, USA). Immunoreactive bands were detected using TOPview™ ECL Pico Plus Western Substrate (Enzynomics, Daejeon, Republic of Korea). For membrane reprobing, blots were stripped with Restore™ Western Blot Stripping Buffer (Thermo Scientific, Waltham, MA, USA). Images were acquired using a ChemiDoc MP™ Imaging System (Bio-Rad, Hercules, CA, USA) and quantified with ImageJ software. Antibody details, including catalog numbers and dilution ratios, are provided in **Supplementary Table 2**.

### 2.10 Immunocytochemistry

For immunocytochemical analysis, BV2 microglial cells were fixed with 4% paraformaldehyde for 10 min at room temperature and washed three times with PBS. Cells were incubated overnight at 4 °C with primary antibodies diluted in PBS containing FBS. Primary antibodies targeted CD11b, phospho-RET, phospho-AKT, phospho-ERK, phospho-NF-κB, phospho-CREB, NLRP3, caspase-1, and microglial state markers (reactive: CD16/32; tissue repair-associated marker: CD206; DAM: Clec7a; and MGnD: Galectin-3); antibody details are provided in **Supplementary Table 2**. CD11b or Iba1 was selectively used as a pan-microglial marker based on primary antibody host compatibility to avoid species overlap during co-immunostaining. Rat-derived CD11b antibody was used for co-staining with rabbit primary antibodies, whereas mouse-derived Iba1 was used for co-staining with CD16/32, CD206, Clec7a, and Galectin-3. After incubation, cells were washed with PBS and incubated with Alexa Fluor 488- or 594-conjugated secondary antibodies (1:200; Invitrogen, Carlsbad, CA, USA) for 2 h at room temperature in the dark. For rabbit primary antibodies against NLRP3 and caspase-1, a FlexAble CoraLite^®^ Plus 488 or 647 Antibody Labeling Kit (Proteintech, Rosemont, IL, USA) was used according to the manufacturer’s instructions. Cells were washed three times with PBS and counterstained with DAPI (Thermo Scientific, Waltham, MA, USA) for 10 min to visualize nuclei. After final washes, samples were mounted with fluorescence mounting medium (Dako, Santa Clara, CA, USA). Fluorescence images were acquired with a DMi8 inverted microscope equipped with the THUNDER imaging system (Leica Microsystems, Wetzlar, Germany) and analyzed with ImageJ software (National Institutes of Health, Bethesda, MD, USA).

### 2.11 Intracellular reactive oxygen species measurement using flow cytometry

Intracellular ROS generation was measured in BV2 microglial cells using flow cytometry. BV2 cells were stimulated with LPS (200 ng/mL) or PBS for 30 min, followed by treatment with Selpercatinib (5 μM) or 1% DMSO vehicle for 23.5 h. After treatment, cells were harvested, and 2 × 10^5^ cells from each experimental condition were incubated in culture medium containing 20 μM 2′,7′-dichlorofluorescin diacetate (DCFDA; Abcam, Cat. No. ab113851, Cambridge, UK) for 30 min at 37 °C. Cells were then gently resuspended and stained with 7-aminoactinomycin D (7-AAD; BioLegend, Cat. No. 420404, San Diego, CA, USA) for 15 min in the dark to exclude dead cells. The samples were subsequently analyzed using a CytoFLEX Flow Cytometer (Beckman Coulter, Brea, CA, USA). Intracellular ROS levels were quantified based on the geometric mean fluorescence intensity (GeoMFI) of DCF-positive cells.

### 2.12 Enzyme-linked immunosorbent assay

IL-1β levels in BV2 microglial cell lysates were measured using a Mouse IL-1β Uncoated ELISA Kit with Plates (Cat. No. 88-7013A-76; Invitrogen, Waltham, MA, USA) according to the manufacturer’s protocol. Briefly, ELISA plates were coated with 100 μL/well of capture antibody solution and incubated overnight at 4 °C. After washing, the wells were blocked with 200 μL/well of ELISA/ELISPOT diluent for 1 h at room temperature. Cell lysates were collected after treatment, and total protein concentrations were determined for normalization. Equal amounts of protein were loaded for each sample, with 50 μg of total protein per well. Standards and samples were added to the designated wells and incubated for 2 h at room temperature on a microplate shaker at 400 rpm. After washing, 100 μL/well of detection antibody solution was added and incubated for 1 h at room temperature with shaking. The wells were then washed and incubated with 100 μL/well of streptavidin-HRP solution for 30 min at room temperature with shaking. Following a final wash, 100 μL/well of TMB substrate solution was added and incubated for 15–30 min at room temperature in the dark. The reaction was stopped by adding 100 μL/well of stop solution, and absorbance was measured at 450 nm using a microplate reader. IL-1β concentrations were calculated from the standard curve and normalized to the total protein amount.

### 2.13 Animals and *in vivo* neuroinflammation model

All animal experiments were conducted in accordance with the IACUC guidelines of Kangwon National University (protocol No. KW-230907-3). Male and female C57BL/6N mice (8–9 weeks old; Samtaco, Osan, Republic of Korea) were housed under controlled conditions (22 ± 2 °C, 50 ± 5% humidity) with a 12-h light/dark cycle and free access to food and water. Mice were randomly assigned to three groups: control, LPS, and LPS + Selpercatinib. Acute neuroinflammation was induced by a single i.p. injection of LPS (10 mg/kg) or PBS. For post-treatment, Selpercatinib (1, 5, or 10 mg/kg, i.p.) or vehicle (10% DMSO, 40% PEG300, and 5% Tween 80 in distilled water) was administered 30 min after LPS injection. Additional Selpercatinib or vehicle doses were given at 2.5- or 8-h intervals. At 2.5 or 8 h after the final administration, mice were deeply anesthetized by intraperitoneal injection of avertin (2.5% v/v, 150 mg/kg) and then euthanized. The hippocampus was rapidly dissected for analysis.

### 2.14 Statistical analysis

Statistical analyses were performed using the GraphPad Prism 9 software (GraphPad Software, San Diego, CA, USA). The n values indicate independent biological replicates. Data were assessed for normality using the Shapiro–Wilk test prior to statistical analysis. Multiple-group comparisons were analyzed by one-way analysis of variance (ANOVA) followed by Tukey’s *post hoc* test. Data are presented as the mean ± standard deviation (SD). Statistical significance was set at p < 0.05. Significance levels are indicated as follows: *p < 0.05, **p < 0.01, ***p < 0.001, and ****p < 0.0001. The exact *p*-values for all comparisons are provided in **Supplementary Table 3**.

## 3 Results

### 3.1 Selpercatinib suppresses RET phosphorylation and proinflammatory responses in LPS-stimulated BV2 microglia

The role of Selpercatinib in regulating microglial neuroinflammatory responses remains unclear. The chemical structure of Selpercatinib is shown in **Figure 1A**. To examine its anti-inflammatory effects, LPS was used to induce microglial activation in BV2 cells *in vitro*. Selpercatinib cytotoxicity was first evaluated by treating BV2 microglia with increasing concentrations (0–10 μM) for 24 h. Cell viability, assessed by CCK-8 assay, was not significantly affected at concentrations up to 5 μM, whereas a slight reduction was observed at 10 μM (**Figure 1B**). Accordingly, 5 μM was selected as the working concentration for subsequent *in vitro* experiments. We further examined cell viability in response to combined LPS and Selpercatinib treatment used in subsequent inflammatory experiments. BV2 microglia were co-treated with LPS (200 ng/mL) and Selpercatinib (5 μM) for 24 h. LPS and Selpercatinib, alone or in combination, did not significantly affect cell viability compared with the control group (**Supplementary Figure 1A**). These findings indicate that the effects of Selpercatinib observed under LPS-stimulated conditions were not associated with a reduction in BV2 cell viability. To determine whether Selpercatinib modulates inflammatory responses in activated microglia, BV2 cells were stimulated with 200 ng/mL LPS and treated with 5 μM Selpercatinib 30 min after LPS exposure. Proinflammatory gene expression was analyzed by quantitative real-time PCR. LPS stimulation increased the mRNA expression of *il-1β, il-6, tnf-α, cox-2, inos, ccl-2*, and *ccl-7*, whereas Selpercatinib attenuated these increases (**Figure 1C**). These results indicate that Selpercatinib suppresses LPS-induced proinflammatory gene expression in microglia.

**Figure 1:**
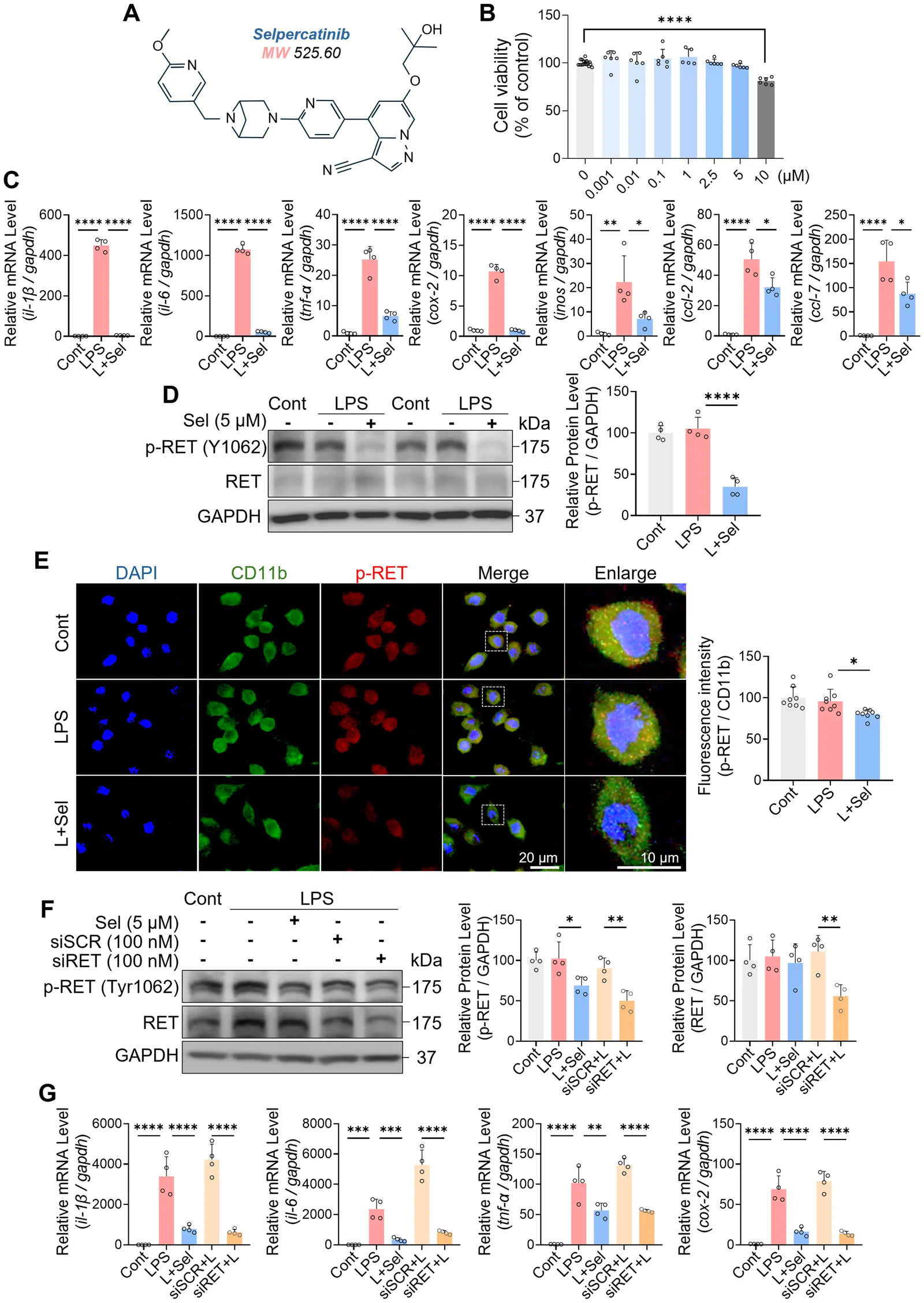
Selpercatinib reduces RET phosphorylation and proinflammatory responses in LPS-stimulated BV2 microglia. **(A)** Chemical structure of Selpercatinib. **(B)** BV2 microglial cell viability after 24 h of Selpercatinib treatment at increasing concentrations, assessed by CCK-8 assay. **(C)** Selpercatinib attenuated LPS-induced mRNA expression of *il-1β, il-6, tnf-α, cox-2, inos, ccl-2*, and *ccl-7* in BV2 microglia. BV2 cells were stimulated with 200 ng/mL LPS and treated with 5 μM Selpercatinib 30 min after LPS exposure (n=4/group). **(D)** Selpercatinib reduced RET phosphorylation at Tyr1062 without altering total RET expression in LPS-treated BV2 microglia (n=4/group). **(E)** Immunocytochemical analysis showed reduced p-RET immunoreactivity in CD11b-positive BV2 microglia after Selpercatinib treatment (n=8/group). **(F)** RET knockdown significantly reduced total RET expression and p-RET (Tyr1062) levels in LPS-stimulated BV2 microglia (n=4/group). **(G)** RET knockdown significantly reduced LPS-induced mRNA expression of *cox-2, il-6, il-1β*, and *tnf-α* in BV2 microglia (n=4/group). Scale bars: 20 μm for merged images and 10 μm for enlarged images. All values are presented as the mean ± SD. *p < 0.05, **p < 0.01, ****p < 0.0001. The n values indicate independent biological replicates. Cont, control; LPS, LPS-treated group; L+Sel, LPS + Selpercatinib; siSCR+L, scrambled control siRNA + LPS; siRET+L, RET-targeting siRNA + LPS.

Selpercatinib is a selective RET kinase inhibitor; however, its role in regulating RET activation during microglial inflammatory responses remains undefined. To assess whether Selpercatinib affects RET signaling, BV2 microglia were stimulated with LPS for 30 min, treated with 5 μM Selpercatinib 15 min after LPS treatment, and analyzed for RET phosphorylation by western blotting. Selpercatinib reduced RET phosphorylation at Tyr1062 compared with LPS treatment without affecting total RET expression (**Figure 1D**). Consistently, immunocytochemical analysis showed that p-RET immunoreactivity colocalized with the microglial marker CD11b was decreased in LPS-stimulated BV2 cells after Selpercatinib treatment (**Figure 1E**). To further characterize RET signaling under LPS-stimulated conditions, we examined specific RET ligand, GDNF secretion and temporal changes in RET phosphorylation following LPS stimulation. Secreted GDNF was detectable under basal conditions in BV2 microglial cells; however, LPS treatment for 45 min, 6 h, or 24 h did not significantly alter GDNF levels (**Supplementary Figure 1B**). Both total RET and p-RET (Tyr1062) were also detectable in untreated BV2 microglia. p-RET levels remained unchanged at 45 min and 6 h after LPS treatment but were significantly decreased after 24 h of LPS stimulation without changes in total RET expression (**Supplementary Figure 1C**). These findings indicate that LPS stimulation did not increase GDNF secretion or RET phosphorylation under the experimental conditions used in this study.

To genetically validate the involvement of RET in LPS-induced inflammatory responses, BV2 microglia were reverse-transfected with RET-targeting siRNA (siRET; 100 nM) or scrambled control siRNA (siSCR; 100 nM) for 48 h. For RET phosphorylation analysis, cells were stimulated with LPS (200 ng/mL) or PBS 45 min before the end of the transfection period; Selpercatinib (5 μM) was added 30 min after LPS stimulation, resulting in a 15 min treatment period. Western blot analysis showed that siRET significantly reduced total RET expression and consequently decreased p-RET (Tyr1062) levels compared with those in the LPS + siSCR group, confirming effective RET knockdown in BV2 microglia (**Figure 1F**).

We next examined whether genetic suppression of RET affects LPS-induced proinflammatory gene expression. BV2 microglia were reverse-transfected with siRET or siSCR for 48 h. Cells were stimulated with LPS (200 ng/mL) or PBS 6 h before the end of the transfection period; Selpercatinib (5 μM) was added 30 min after LPS stimulation for the remaining 5.5 h. Quantitative real-time PCR analysis showed that RET knockdown significantly reduced the LPS-induced mRNA expression of *il-1β, il-6, tnf-α*, and *cox-2*, compared with those in the LPS + siSCR group (**Figure 1G**). These inhibitory effects were comparable to those observed following Selpercatinib treatment. Together, these findings demonstrate that both pharmacological RET inhibition by Selpercatinib and genetic RET knockdown attenuate LPS-induced proinflammatory responses in BV2 microglia, supporting the involvement of RET signaling in microglia-mediated neuroinflammation.

### 3.2 Selpercatinib attenuates AKT and ERK signaling in LPS-stimulated BV2 microglia

To further define downstream pathways affected by RET inhibition, we examined the effects of Selpercatinib on AKT and ERK activation in LPS-stimulated BV2 microglia. To test whether Selpercatinib affects AKT signaling, BV2 microglia were stimulated with LPS for 45 min, treated with 5 μM Selpercatinib 45 min after LPS treatment, and western blotting was conducted. The results showed that LPS stimulation increased AKT phosphorylation at Ser473, whereas Selpercatinib significantly reduced p-AKT levels without altering total AKT expression (**Figure 2A**). Consistently, immunocytochemical analysis showed increased p-AKT immunoreactivity in CD11b-positive microglia after LPS stimulation, whereas Selpercatinib attenuated this increase (**Figure 2B**).

**Figure 2:**
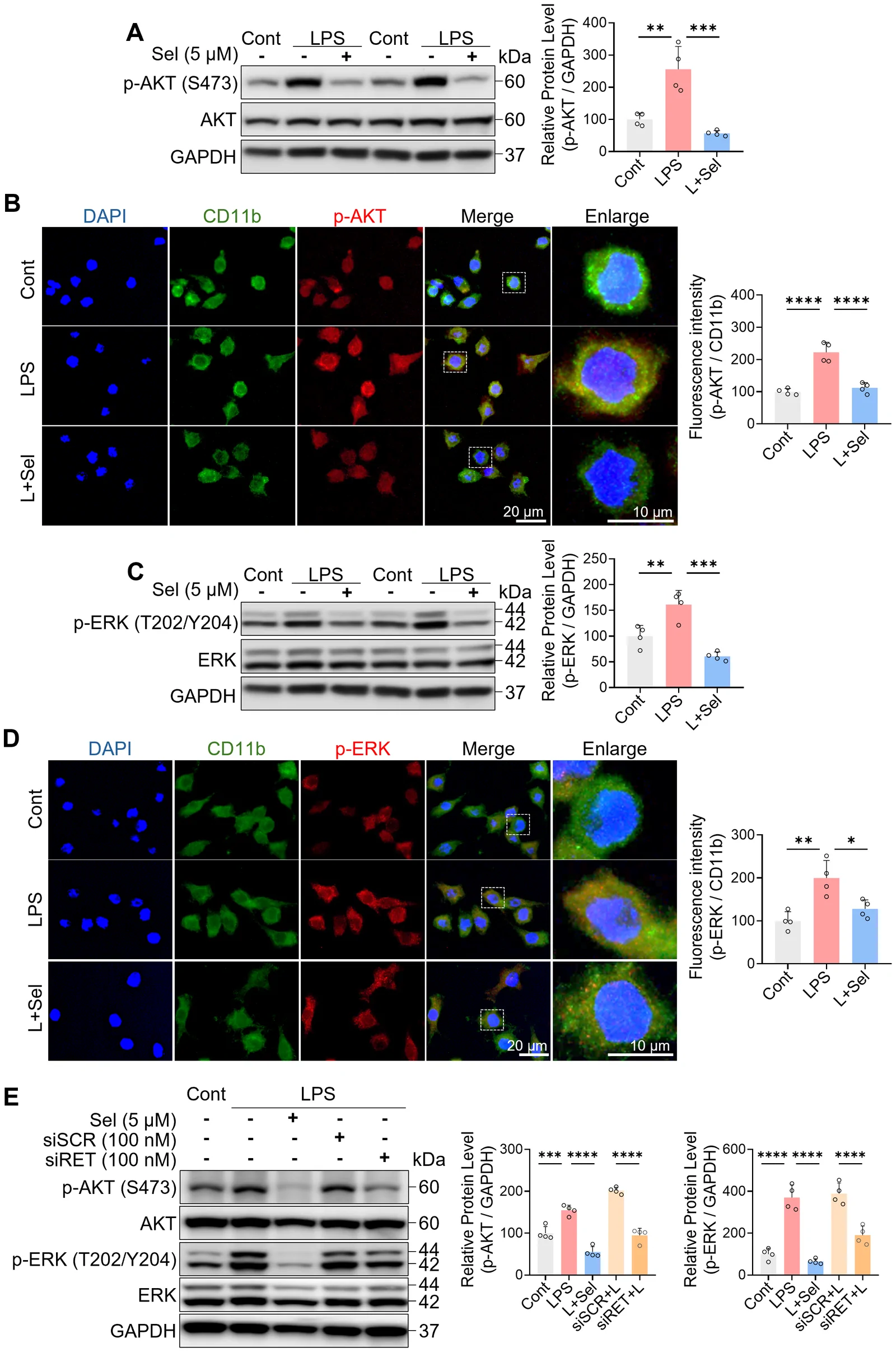
Selpercatinib attenuates AKT and ERK signaling in LPS-stimulated BV2 microglia. **(A)** Selpercatinib reduced LPS-induced AKT phosphorylation at Ser473 without altering total AKT expression in BV2 microglia (n=4/group). **(B)** Immunocytochemical analysis showed reduced p-AKT immunoreactivity in CD11b-positive BV2 microglia after Selpercatinib treatment (n=4/group). **(C)** Selpercatinib reduced LPS-induced ERK phosphorylation at Thr202/Tyr204 without altering total ERK expression (n=4/group). **(D)** Immunocytochemical analysis showed reduced p-ERK immunoreactivity in CD11b-positive BV2 microglia after Selpercatinib treatment. BV2 cells were stimulated with 200 ng/mL LPS and treated with 5 μM Selpercatinib after LPS exposure (n=4/group). **(E)** RET knockdown significantly reduced LPS-induced AKT phosphorylation at Ser473 and ERK phosphorylation at Thr202/Tyr204 without altering total AKT or ERK expression (n=4/group). Scale bars: 20 μm for merged images and 10 μm for enlarged images. All values are presented as the mean ± SD. *p < 0.05, **p < 0.01, ***p < 0.001, ****p < 0.0001. The n values indicate independent biological replicates. Cont, control; LPS, LPS-treated group; L+Sel, LPS + Selpercatinib; siSCR+L, scrambled control siRNA + LPS; siRET+L, RET-targeting siRNA + LPS.

We next evaluated the effect of Selpercatinib on ERK signaling. LPS stimulation increased ERK phosphorylation at Thr202/Tyr204, whereas Selpercatinib suppressed p-ERK levels without altering total ERK expression (**Figure 2C**). Similarly, immunocytochemical analysis showed increased p-ERK immunoreactivity in LPS-treated BV2 microglia, which was reduced after Selpercatinib treatment, showing decreased p-ERK intensity in the presence of Selpercatinib (**Figure 2D**). To determine whether genetic suppression of RET similarly affects AKT and ERK signaling, BV2 microglia were reverse-transfected with siRET (100 nM) or siSCR (100 nM) for 48 h. Cells were stimulated with LPS (200 ng/mL) or PBS 1.5 h before the end of the transfection period; Selpercatinib (5 μM) was added 45 min after LPS stimulation, resulting in a 45-min treatment period. Western blot analysis showed that RET knockdown significantly reduced LPS-induced AKT phosphorylation at Ser473 and ERK phosphorylation at Thr202/Tyr204 compared with those in the LPS + siSCR group, without altering total AKT or ERK expression (**Figure 2E**). These effects were consistent with those observed following Selpercatinib treatment. Together, these results indicate that both pharmacological inhibition and genetic suppression of RET attenuate LPS-induced AKT and ERK activation, supporting RET as an upstream regulator of these inflammatory signaling pathways in BV2 microglia.

### 3.3 Selpercatinib inhibits LPS-induced NF-κB and CREB activation in BV2 microglia

NF-κB and CREB are major transcriptional regulators of microglial inflammatory activation and are functionally linked to upstream AKT and ERK signaling pathways (33–35). After showing that Selpercatinib reduces RET phosphorylation and attenuates downstream AKT and ERK phosphorylation in BV2 microglia, we next examined whether Selpercatinib modulates NF-κB and CREB activation. BV2 microglia were stimulated with 200 ng/mL LPS for 30 min and then treated with 5 μM Selpercatinib. After 5.5 h, nuclear fractions were prepared, and NF-κB phosphorylation was analyzed by western blotting. LPS stimulation increased nuclear p-NF-κB (Ser536) levels compared with control cells, whereas Selpercatinib reduced LPS-induced nuclear p-NF-κB expression (**Figure 3A**). Total NF-κB levels in the nuclear fraction were not significantly altered in the LPS and LPS + Selpercatinib groups, and histone H3 was used as a nuclear loading control (**Figure 3A**). Consistently, immunocytochemical analysis showed increased p-NF-κB immunoreactivity in CD11b-positive BV2 microglia after LPS stimulation. LPS also induced p-NF-κB accumulation in the nuclear region, as shown by colocalization with DAPI-stained nuclei. Selpercatinib reduced LPS-induced p-NF-κB immunoreactivity (**Figure 3B**). These results suggest that Selpercatinib suppresses LPS-induced NF-κB activation and nuclear translocation in BV2 microglia.

**Figure 3:**
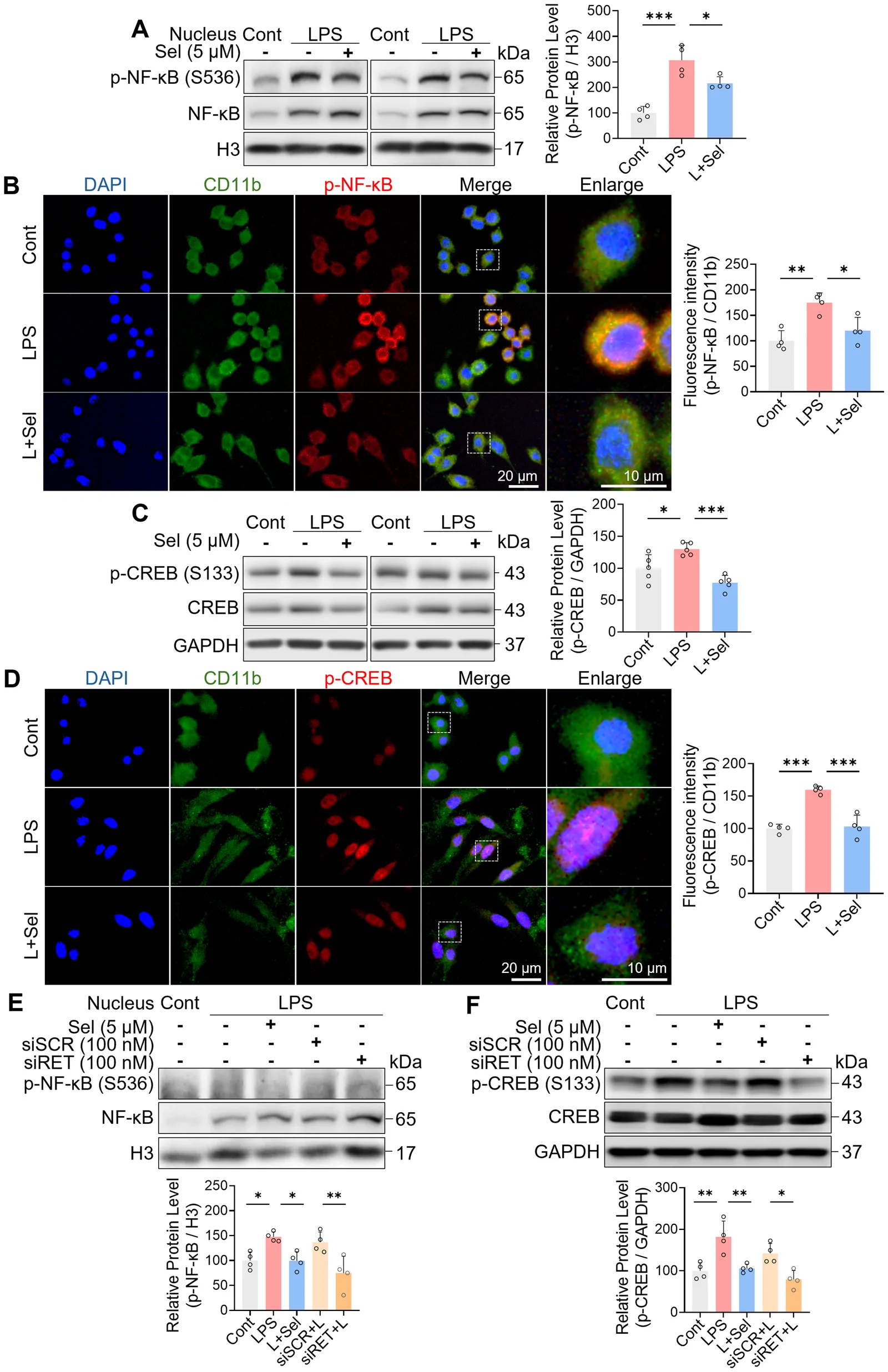
Selpercatinib inhibits LPS-induced NF-κB and CREB activation in BV2 microglia. **(A)** Selpercatinib reduced LPS-induced nuclear p-NF-κB (Ser536) levels in BV2 microglia. BV2 cells were stimulated with 200 ng/mL LPS for 30 min and then treated with 5 μM Selpercatinib for 5.5 h, and nuclear fractions were analyzed by western blotting. Histone H3 was used as a nuclear loading control (n=4/group). **(B)** Immunocytochemical analysis showed reduced LPS-induced p-NF-κB immunoreactivity and nuclear accumulation in CD11b-positive BV2 microglia after Selpercatinib treatment (n=4/group). **(C)** Selpercatinib reduced LPS-induced CREB phosphorylation at Ser133 without altering total CREB expression. BV2 cells were stimulated with 200 ng/mL LPS and collected 2.5 h after LPS treatment for western blotting (n=5/group). **(D)** Immunocytochemical analysis showed reduced p-CREB immunoreactivity in CD11b-positive BV2 microglia after Selpercatinib treatment (n=4/group). **(E)** RET knockdown significantly reduced LPS-induced nuclear p-NF-κB (Ser536) levels in BV2 microglia (n=4/group). **(F)** RET knockdown significantly reduced LPS-induced CREB phosphorylation at Ser133 without altering total CREB expression (n=4/group). Scale bars: 20 μm for merged images and 10 μm for enlarged images. All values are presented as the mean ± SD. *p < 0.05, **p < 0.01, ***p < 0.001. The n values indicate independent biological replicates. Cont, control; LPS, LPS-treated group; L+Sel, LPS + Selpercatinib; siSCR+L, scrambled control siRNA + LPS; siRET+L, RET-targeting siRNA + LPS.

We next investigated whether Selpercatinib modulates CREB phosphorylation in LPS-stimulated BV2 microglia. BV2 cells were stimulated with 200 ng/mL LPS for 30 min and collected 2.5 h after LPS treatment, after which CREB phosphorylation was assessed by western blotting. LPS stimulation increased CREB phosphorylation at Ser133, whereas Selpercatinib attenuated this increase without altering total CREB expression (**Figure 3C**). Immunocytochemical analysis showed increased p-CREB immunoreactivity in CD11b-positive microglia after LPS stimulation, whereas Selpercatinib reduced this LPS-induced increase (**Figure 3D**). Collectively, these results demonstrate that Selpercatinib suppresses LPS-induced NF-κB nuclear translocation and CREB phosphorylation in BV2 microglia, suggesting that inhibition of these transcriptional pathways may contribute to Selpercatinib-mediated suppression of inflammatory signaling downstream of RET, AKT, and ERK signaling.

To further assess whether RET contributes to the regulation of downstream NF-κB and CREB signaling, BV2 microglia were reverse-transfected with siRET (100 nM) or siSCR (100 nM) for 48 h. For NF-κB analysis, cells were stimulated with LPS (200 ng/mL) or PBS 6 h before the end of the transfection period; Selpercatinib (5 μM) was added 30 min after LPS stimulation for the remaining 5.5 h. Nuclear fractions were subsequently prepared, and p-NF-κB levels were analyzed by western blotting. RET knockdown significantly reduced LPS-induced nuclear p-NF-κB (Ser536) levels compared with those in the LPS + siSCR group (**Figure 3E**), indicating that genetic suppression of RET attenuates NF-κB activation and nuclear accumulation. For CREB analysis, BV2 microglia were reverse-transfected with siRET or siSCR for 48 h. Cells were stimulated with LPS (200 ng/mL) or PBS 3 h before the end of the transfection period; Selpercatinib (5 μM) was added 30 min after LPS stimulation for the remaining 2.5 h. Western blot analysis showed that RET knockdown significantly reduced LPS-induced CREB phosphorylation at Ser133 compared with those in the LPS + siSCR group, without altering total CREB expression (**Figure 3F**). These effects were consistent with those observed following Selpercatinib treatment. Collectively, the consistent effects of Selpercatinib treatment and RET knockdown support the involvement of RET in AKT/ERK–NF-κB/CREB inflammatory signaling in LPS-stimulated BV2 microglia.

### 3.4 Selpercatinib modulates microglial phenotypic marker expression and reduces ROS generation in LPS-stimulated BV2 microglia

Microglial activation involves dynamic changes in phenotypic marker expression, including reactive, protective, DAM-related, and microglial neurodegenerative phenotype (MGnD)-related markers (36). After showing that Selpercatinib inhibits LPS-induced inflammatory signaling pathways, we next examined whether Selpercatinib affects microglial phenotype marker expression in LPS-stimulated BV2 microglia. BV2 cells were stimulated with 200 ng/mL LPS for 30 min and then treated with 5 μM Selpercatinib. After 23.5 h, immunocytochemical analysis assessed expression of the reactive microglial marker CD16/32, tissue repair-associated marker CD206, DAM marker Clec7a, and MGnD marker Galectin-3. LPS stimulation increased CD16/32 immunoreactivity in Iba1-positive BV2 microglia compared with control cells, indicating reactive microglial activation. Selpercatinib reduced LPS-induced CD16/32 fluorescence intensity (**Figures 4A, B**). Conversely, CD206 immunoreactivity was not increased by LPS stimulation alone, whereas Selpercatinib enhanced CD206 expression in LPS-stimulated BV2 microglia (**Figures 4A, C**). These results suggest that Selpercatinib attenuates acquisition of a reactive microglial phenotype while promoting CD206 expression under neuroinflammatory conditions.

**Figure 4:**
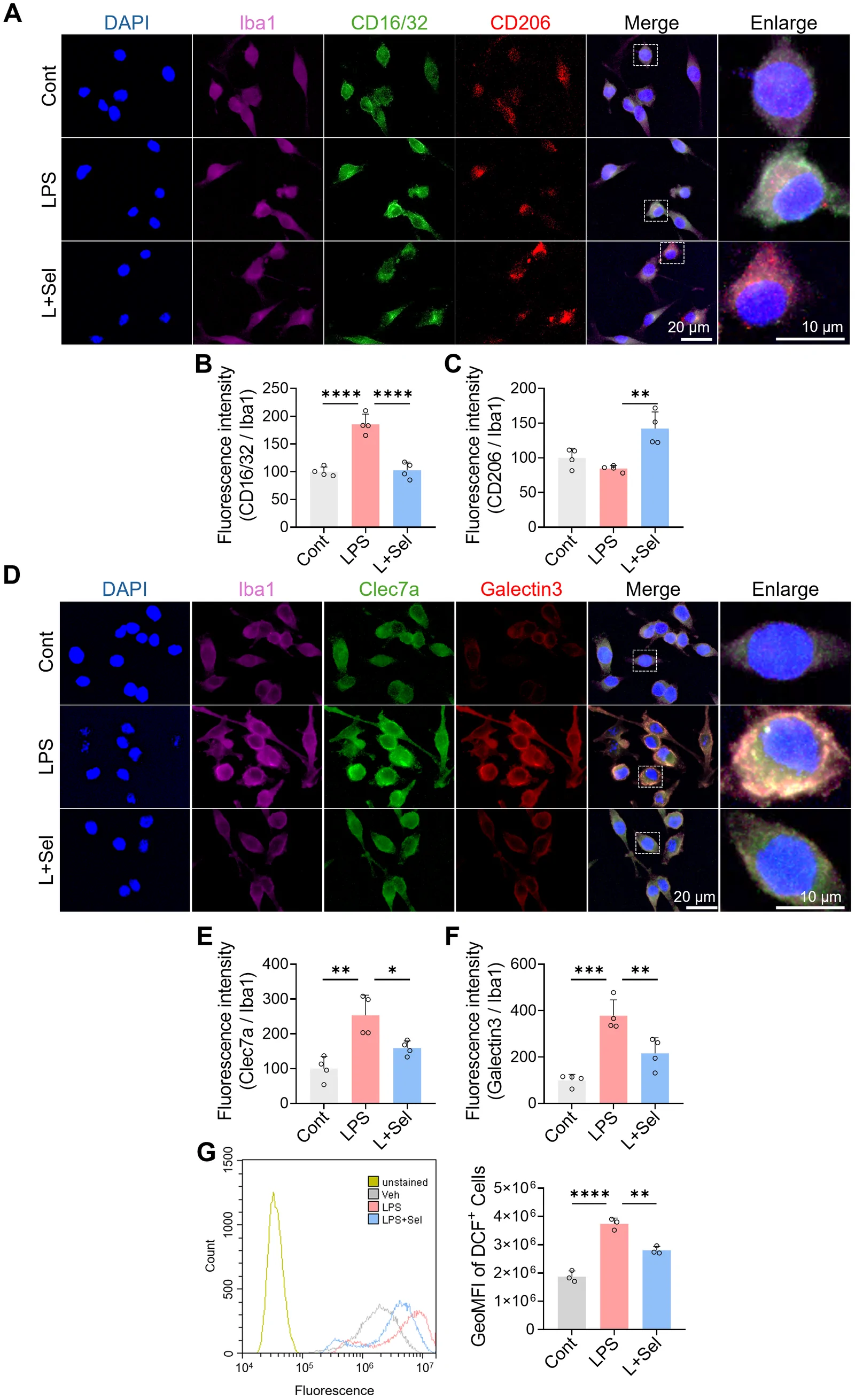
Selpercatinib modulates microglial activation marker expression and reduces ROS generation in LPS-stimulated BV2 microglia. **(A)** Representative immunocytochemical images showing CD16/32 and CD206 expression in Iba1-positive BV2 microglia. BV2 cells were stimulated with 200 ng/mL LPS for 30 min and then treated with 5 μM Selpercatinib for 23.5 h. **(B)** Quantification of CD16/32 fluorescence intensity showed that Selpercatinib reduced LPS-induced CD16/32 expression in Iba1-positive BV2 microglia (n=4/group). **(C)** Quantification of CD206 fluorescence intensity showed that Selpercatinib increased CD206 expression in LPS-stimulated BV2 microglia (n=4/group). **(D)** Representative immunocytochemical images showing Clec7a and Galectin-3 expression in Iba1-positive BV2 microglia. **(E)** Quantification of Clec7a fluorescence intensity showed that Selpercatinib reduced LPS-induced Clec7a expression in Iba1-positive BV2 microglia (n=4/group). **(F)** Quantification of Galectin-3 fluorescence intensity showed that Selpercatinib reduced LPS-induced Galectin-3 expression in Iba1-positive BV2 microglia (n=4/group). **(G)** Flow cytometric analysis of intracellular ROS generation showed that LPS stimulation significantly increased DCF fluorescence, whereas Selpercatinib reduced LPS-induced ROS generation in BV2 microglia (n=3/group). Scale bars: 20 μm for merged images and 10 μm for enlarged images. All values are presented as the mean ± SD. *p < 0.05, **p < 0.01, ***p < 0.001, ****p < 0.0001. The n values indicate independent biological replicates. Cont, control; LPS, LPS-treated group; L+Sel, LPS + Selpercatinib.

We next examined Clec7a and Galectin-3 expression, which are associated with DAM and MGnD-like microglial states, respectively (25, 26, 29). LPS stimulation increased Clec7a immunoreactivity in Iba1-positive microglia, and Selpercatinib attenuated this increase (**Figures 4D, E**). Similarly, Galectin-3 expression increased after LPS stimulation, whereas Selpercatinib reduced LPS-induced Galectin-3 fluorescence intensity (**Figures 4D, F**). Collectively, these results demonstrate that Selpercatinib modulates LPS-induced microglial marker expression patterns by suppressing reactive, DAM-, and MGnD-associated marker expression while enhancing CD206 in BV2 microglia.

To further evaluate the functional inflammatory response of microglia, intracellular ROS generation was assessed using flow cytometry. BV2 microglia were stimulated with LPS (200 ng/mL) or PBS; Selpercatinib (5 μM) was added 30 min after LPS stimulation. Cells were harvested after 23.5 h of Selpercatinib treatment and analyzed for intracellular ROS generation through DCF fluorescence. LPS stimulation significantly increased the GeoMFI of DCF-positive cells compared with that of the control group, whereas Selpercatinib significantly reduced LPS-induced ROS generation (**Figure 4G**). These results provide additional functional evidence that Selpercatinib attenuates LPS-induced inflammatory activation in BV2 microglia. Collectively, our findings demonstrate that Selpercatinib modulates LPS-induced microglial activation by suppressing reactive, DAM-, and MGnD-associated marker expression, enhancing CD206 expression, and reducing intracellular ROS generation in BV2 microglia.

### 3.5 Selpercatinib inhibits LPS-induced inflammasome activation in BV2 microglia

Inflammasome activation is a central downstream event in microglia-mediated inflammatory responses and contributes to proinflammatory cytokine maturation (37, 38). Because Selpercatinib attenuated LPS-induced inflammatory signaling and microglial marker expression patterns, we next investigated whether Selpercatinib modulates inflammasome activation in BV2 microglia. BV2 cells were stimulated with 200 ng/mL LPS for 30 min and then treated with 5 μM Selpercatinib. After 23.5 h, inflammasome-related protein levels were examined by western blotting. LPS stimulation increased NLRP3 and ASC protein levels compared with control cells, whereas Selpercatinib reduced these LPS-induced increases (**Figures 5A, B**). In addition, LPS elevated pro-caspase-1 and cleaved caspase-1 levels, indicating activation of caspase-1-dependent inflammasome signaling. Selpercatinib attenuated both pro-caspase-1 and cleaved caspase-1 expression in LPS-stimulated BV2 microglia (**Figures 5A, B**). We further examined GSDMD, a downstream marker associated with inflammasome-mediated pyroptotic activation. LPS stimulation increased full-length GSDMD (GSDMD-F) expression, whereas Selpercatinib suppressed this increase (**Figures 5A, B**). Consistent with these changes in inflammasome-related proteins, ELISA analysis showed that LPS significantly increased IL-1β protein levels, whereas Selpercatinib significantly reduced LPS-induced IL-1β production in BV2 microglia (**Figure 5C**). These findings suggest that early post-LPS treatment with Selpercatinib attenuates the development of NLRP3 inflammasome activation in BV2 microglia.

**Figure 5:**
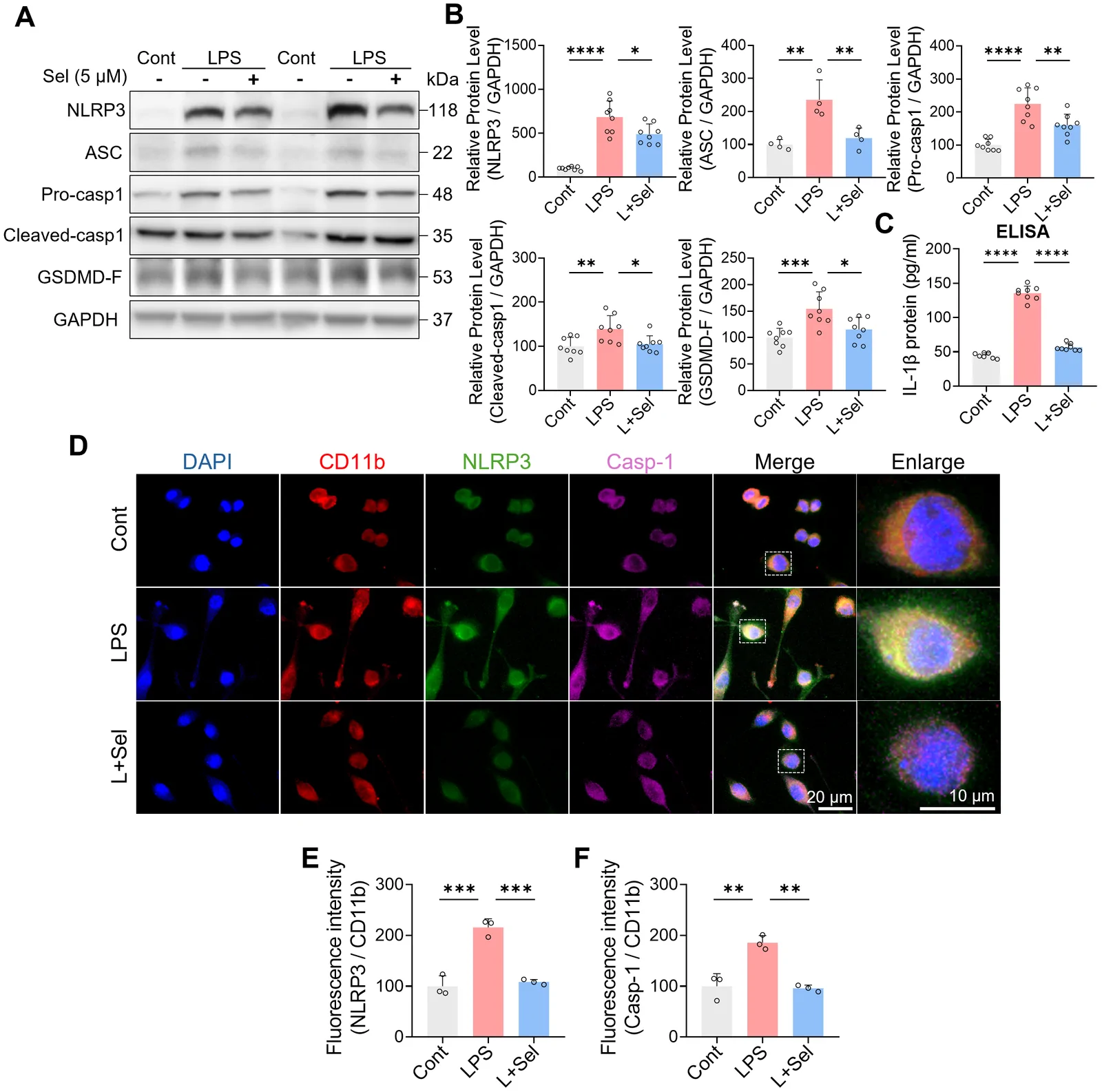
Selpercatinib inhibits LPS-induced inflammasome activation in BV2 microglia. **(A)** Selpercatinib reduced LPS-induced inflammasome-related protein expression, including NLRP3, ASC, pro-caspase-1, cleaved caspase-1, and full-length GSDMD (GSDMD-F), in BV2 microglia. BV2 cells were stimulated with 200 ng/mL LPS for 30 min and then treated with 5 μM Selpercatinib for 23.5 h. **(B)** Quantification of inflammasome-related protein levels showed that Selpercatinib reduced LPS-induced increases in NLRP3, ASC, pro-caspase-1, cleaved caspase-1, and GSDMD-F expression (n=4-8/group). **(C)** ELISA analysis showed that LPS markedly increased IL-1β protein levels in BV2 cell lysates, whereas Selpercatinib significantly reduced LPS-induced IL-1β production (n=7-8/group). **(D)** Representative immunocytochemical images showing NLRP3 and caspase-1 expression in CD11b-positive BV2 microglia. **(E, F)** Quantitative fluorescence analysis showed that Selpercatinib reduced LPS-induced NLRP3 and caspase-1 immunoreactivity in CD11b-positive BV2 microglia (n=3/group). Scale bars: 20 μm for merged images and 10 μm for enlarged images. All values are presented as the mean ± SD. *p < 0.05, **p < 0.01, ***p < 0.001, ****p < 0.0001. The n values indicate independent biological replicates. Cont, control; LPS, LPS-treated group; L+Sel, LPS + Selpercatinib.

Consistently, immunocytochemical analysis showed increased NLRP3 and caspase-1 immunoreactivity in CD11b-positive BV2 microglia after LPS stimulation (**Figure 5D**). Selpercatinib reduced the LPS-induced increases in NLRP3 and caspase-1 fluorescence intensity (**Figures 5D–F**). Quantitative analysis confirmed increased NLRP3 and caspase-1 immunoreactivities after LPS stimulation and decreased immunoreactivities after Selpercatinib treatment (**Figures 5E, F**). Collectively, these results demonstrate that early post-LPS treatment with Selpercatinib attenuates subsequent inflammasome activation in BV2 microglia.

### 3.6 Selpercatinib attenuates LPS-induced hippocampal inflammation and inflammasome activation *in vivo*

To determine whether the anti-neuroinflammatory effects of Selpercatinib observed in BV2 microglia are reproduced under *in vivo* inflammatory conditions, we examined Selpercatinib in an LPS-induced acute systemic inflammation mouse model. Mice were intraperitoneally (i.p.) injected with LPS at 10 mg/kg, followed by three Selpercatinib injections at 10 mg/kg. To assess proinflammatory gene expression, Selpercatinib was administered at 2.5-h intervals after LPS injection, and hippocampal tissues were collected 8 h after LPS administration (**Figure 6A**). Quantitative real-time PCR analysis showed that LPS increased hippocampal mRNA expression of *il-1β* compared with control mice, whereas Selpercatinib reduced this LPS-induced increase (**Figure 6B**). These results indicate that Selpercatinib attenuates LPS-induced proinflammatory cytokine expression in the hippocampus.

**Figure 6:**
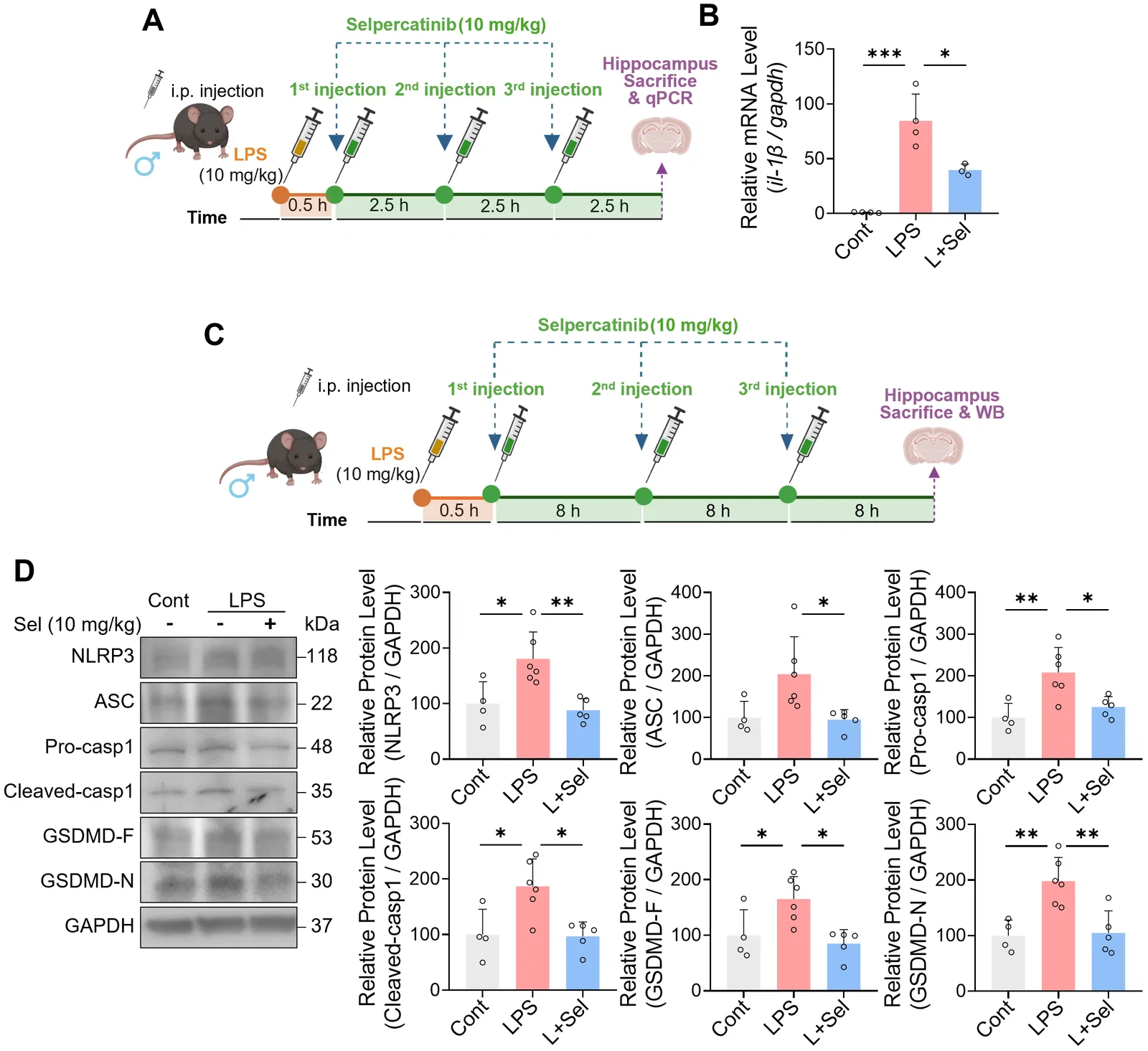
Selpercatinib attenuates LPS-induced hippocampal inflammation and inflammasome activation *in vivo*. **(A)** Schematic of the *in vivo* experimental protocol for hippocampal cytokine analysis. Mice were intraperitoneally injected with LPS at 10 mg/kg, followed by Selpercatinib administration at 10 mg/kg at 2.5-h intervals after LPS injection. Hippocampal tissues were collected 8 h after LPS administration. **(B)** Selpercatinib attenuated LPS-induced mRNA expression of *il-1β* in the hippocampus (n=4/group). **(C)** Schematic of the *in vivo* experimental protocol for hippocampal inflammasome analysis. Mice were intraperitoneally injected with LPS at 10 mg/kg, followed by Selpercatinib administration at 10 mg/kg at 8-h intervals after LPS injection. Hippocampal tissues were collected 24.5 h after LPS administration. **(D)** Selpercatinib reduced LPS-induced inflammasome-related protein expression, including NLRP3, ASC, pro-caspase-1, cleaved caspase-1, full-length GSDMD (GSDMD-F), and N-terminal GSDMD (GSDMD-N), in the hippocampus (n=4-6/group). All values are presented as the mean ± SD. *p < 0.05, **p < 0.01, ***p < 0.001, ****p < 0.0001. The n values indicate independent biological replicates. Cont, control; LPS, LPS-treated group; L+Sel, LPS + Selpercatinib.

We next examined whether Selpercatinib modulates inflammasome activation in the hippocampus after LPS challenge. For this experiment, mice were i.p. injected with LPS at 10 mg/kg, followed by three Selpercatinib injections at 10 mg/kg administered at 8 h intervals after LPS injection. Hippocampal tissues were collected 24.5 h after LPS administration, and inflammasome-related protein expression was analyzed by western blotting (**Figure 6C**). LPS injection increased hippocampal protein levels of NLRP3, pro-caspase-1, cleaved caspase-1, GSDMD-F, and N-terminal GSDMD (GSDMD-N) compared with control mice (**Figure 6D**). Selpercatinib reduced the LPS-induced increases in these inflammasome-related proteins including NLRP3, ASC, pro-caspase-1, cleaved caspase-1 and GSDMD-F and N levels, suggesting attenuation of inflammasome activation *in vivo* (**Figure 6D**).

To further examine the effects of lower Selpercatinib doses, we performed an additional dose-ranging analysis in male mice using Selpercatinib at 1 and 5 mg/kg under the same LPS-induced acute neuroinflammation paradigm (**Supplementary Figure 2A**). Hippocampal western blot analysis showed that 1 mg/kg Selpercatinib significantly reduced GSDMD-N levels, whereas 5 mg/kg significantly reduced GSDMD-F levels compared with those in the LPS group. Other inflammasome-related proteins showed decreasing trends, but the difference did not reach statistical significance at these lower doses (**Supplementary Figure 2B**). These findings indicate partial and component-specific effects of lower Selpercatinib doses on hippocampal inflammasome signaling. In addition, to assess whether the effects of Selpercatinib were also observed in female mice, we examined LPS-induced inflammasome responses following treatment with 10 mg/kg Selpercatinib (**Supplementary Figure 3A**). Selpercatinib significantly reduced NLRP3, ASC, pro-caspase-1, cleaved caspase-1, GSDMD-F, and GSDMD-N levels compared with the levels in the LPS group in the hippocampus of female mice (**Supplementary Figure 3B**). These findings indicate that the inhibitory effects of Selpercatinib on LPS-induced inflammasome-associated responses are also observed in female mice. Collectively, our findings demonstrate that early post-LPS administration of Selpercatinib attenuates the development of hippocampal inflammatory and NLRP3 inflammasome-associated responses *in vivo.* These effects were further supported by partial responses at lower doses and by comparable inflammasome-associated changes in female mice.

## 4 Discussion

This study shows that Selpercatinib attenuates neuroinflammatory responses in LPS-stimulated BV2 microglia and an LPS-induced acute neuroinflammation mouse model. Selpercatinib reduced RET phosphorylation in LPS-stimulated BV2 microglia and reduced multiple proinflammatory mediators, including *il-1β, il-6, tnf-α, cox-2, inos, ccl-2*, and *ccl-7*, in BV2 microglia. Mechanistically, Selpercatinib reduced AKT and ERK signaling activation, inhibited NF-κB nuclear accumulation and CREB phosphorylation, and modulated microglial marker expression under inflammatory conditions. Selpercatinib also inhibited NLRP3 inflammasome activation in BV2 microglia, with reduced expression of NLRP3, ASC, pro-caspase-1, cleaved caspase-1, and GSDMD. These anti-inflammatory effects were also observed *in vivo*; systemic Selpercatinib administration attenuated LPS-induced *il-1β* mRNA expression and suppressed inflammasome-related protein expression in the hippocampus. Together, these findings indicate that early post-LPS treatment with Selpercatinib attenuates the development of microglia-mediated neuroinflammatory responses through modulation of RET-associated inflammatory signaling and NLRP3 inflammasome activation.

A major finding of this study is that Selpercatinib reduced RET phosphorylation at Tyr1062 in LPS-stimulated BV2 microglia, with concurrent suppression of downstream AKT/ERK signaling and proinflammatory cytokine expression, thereby disrupting a signaling module that promotes microglia-mediated neuroinflammation (**Figures 1**, **2**). RET is a receptor tyrosine kinase essential for neural development, neuronal survival, differentiation, and tissue homeostasis through activation by GDNF family ligands and their co-receptors (3, 4). Tyr1062 is a major multi-docking autophosphorylation site within RET that recruits adaptor proteins and couples RET activation to downstream pathways, including PI3K/AKT, RAS/ERK, and other survival- and inflammation-associated cascades (3, 39). Although RET signaling has been extensively studied in developmental biology and RET-driven cancers, its contribution to microglial inflammatory signaling remains poorly defined. A previous study reported that pralsetinib, a RET inhibitor, reduced the expression of inflammatory cytokines such as IL-5 and IL-13, which are major pathological factors in asthma, in an ovalbumin (OVA)-induced mouse model of asthma (40). These findings suggest that RET signaling may be involved in regulating the production of inflammatory mediators. The reduction in p-RET (Tyr1062) in Selpercatinib-treated microglia indicates pharmacological suppression of this pathway under LPS-challenged conditions, thereby restraining AKT and ERK activation and limiting transcriptional induction of inflammatory mediators such as *il-1β, il-6, tnf-α, cox-2, inos, ccl-2*, and *ccl-7*. Importantly, this pharmacological evidence was further supported by genetic RET knockdown. siRET led to a significant reduction in LPS-induced proinflammatory gene expression and attenuation of AKT and ERK phosphorylation, recapitulating the effects observed following Selpercatinib treatment. These findings provide genetic evidence that RET contributes to LPS-induced inflammatory signaling in BV2 microglia and strengthen the mechanistic link between Selpercatinib-mediated RET inhibition and its anti-inflammatory effects. Notably, these effects were observed despite the absence of detectable LPS-induced RET activation. One plausible mechanism is that LPS/TLR4 and RET signaling converge on common downstream pathways, including AKT and ERK, without requiring direct ligand-dependent RET activation. Thus, basal RET-associated signaling may contribute to the modulation of LPS-induced inflammatory responses through these shared downstream signaling pathways.

Consistent with reduced AKT and ERK signaling, Selpercatinib also inhibited NF-κB and CREB activation, two transcriptional regulators that act as central downstream effectors of inflammatory signaling (**Figure 3**). NF-κB is a canonical transcription factor activated by LPS/TLR4 signaling and regulates numerous inflammatory mediators, including *il-1β, il-6, tnf-α, cox-2*, and *inos* (14, 41). AKT and ERK signaling pathways have both been implicated in amplifying NF-κB-dependent transcriptional responses and nuclear translocation (14, 42). In addition, CREB is activated by multiple kinase pathways, including ERK- and AKT-associated signaling modules, and contributes to inflammatory and stress-responsive gene programs in microglia (43). Previous studies reported that NVP-AST487, a chemical inhibitor of RET, reduced AKT activity in the breast cancer cell line J110 (44). In addition, a study using thyroid cancer cell lines (TPC-1, TT, and MZ-CRC-1) showed that treatment with NVP-AST487 decreased AKT phosphorylation (45). Furthermore, in TT cells derived from medullary thyroid carcinoma harboring RET mutations, suppression of RET expression using shRNA was reported to reduce NF-κB transcriptional activity while also inhibiting ERK/MAPK signaling (46). More recently, reduced phosphorylation of CREB was reported in hematopoietic stem cells derived from RET-deficient mice (47). Importantly, genetic suppression of RET produced effects consistent with those of Selpercatinib treatment on these downstream transcriptional regulators, with significantly reduced LPS-induced nuclear p-NF-κB (Ser536) levels and CREB phosphorylation at Ser133, further supporting the involvement of RET in the regulation of NF-κB and CREB activation under inflammatory conditions. Together with the observed reductions in AKT and ERK phosphorylation, these findings support a model in which Selpercatinib attenuates inflammatory responses, at least in substantial part, through inhibition of RET-associated AKT/ERK–NF-κB/CREB signaling.

Another key finding is that Selpercatinib modulated LPS-induced microglial activation marker expression (**Figure 4**). Microglia display marked functional plasticity in response to pathological stimuli, and inflammatory activation is accompanied by changes in markers associated with reactive, protective, DAM-like, and MGnD-like states (36, 48). LPS stimulation increased CD16/32 expression, a marker associated with reactive inflammatory microglia, as well as Clec7a and Galectin-3, which have been linked to DAM- and MGnD-like activation programs in neurodegenerative and inflammatory conditions (27, 29). However, no studies have investigated the pathological characteristics of disease-associated microglia or neurodegeneration-associated microglia following RET inhibition. In this study, Selpercatinib reduced the LPS-induced increases in CD16/32, Clec7a, and Galectin-3 immunoreactivity, indicating that RET inhibition attenuates LPS-induced inflammatory marker expression. In contrast, Selpercatinib increased CD206 expression in LPS-stimulated BV2 microglia. The coordinated reduction in reactive, DAM-, and MGnD-associated markers, together with increased CD206, supports the possibility that Selpercatinib promotes a less inflammatory microglial marker profile by inhibiting RET-dependent signaling after LPS stimulation. Further, Selpercatinib significantly reduced LPS-induced intracellular ROS generation. Because increased ROS generation represents a functional response associated with inflammatory microglial activation, this finding provides additional evidence that Selpercatinib attenuates the functional inflammatory response of LPS-stimulated BV2 microglia.

CD206 is associated with tissue repair and inflammation-resolving microglial responses; however, its expression alone does not define a discrete functional microglial phenotype, and its interpretation depends on the disease context and cellular environment (30, 49). Microglial activation is increasingly recognized as a continuum of heterogeneous and context-dependent states rather than as a binary polarization. Therefore, the increased CD206 expression observed following Selpercatinib treatment should not be interpreted as a complete phenotypic conversion but rather as a shift in the pattern of microglial activation markers under inflammatory conditions. Further multidimensional characterization using broader molecular and functional profiles will be required to define the specific microglial states associated with Selpercatinib treatment.

Beyond suppressing inflammatory signaling and microglial activation marker expression, Selpercatinib inhibited NLRP3 inflammasome activation *in vitro* and *in vivo* (**Figures 5**, **6**). The NLRP3 inflammasome is a major innate immune complex that senses pathogen- and danger-associated signals and promotes caspase-1 activation, IL-1β maturation, and GSDMD-mediated inflammatory responses (17, 22, 50). NLRP3 inflammasome activation generally requires a priming signal, often mediated by NF-κB-dependent transcriptional induction of NLRP3 and IL-1β, followed by an activation step that promotes inflammasome assembly, caspase-1 cleavage, and GSDMD processing (17, 51). In LPS-stimulated BV2 microglia, Selpercatinib reduced NLRP3, ASC, pro-caspase-1, cleaved caspase-1, and GSDMD-F expression, and immunocytochemical analysis confirmed reduced NLRP3 and caspase-1 immunoreactivity in CD11b-positive microglia. Consistent with these *in vitro* findings, systemic Selpercatinib administration attenuated LPS-induced hippocampal *il-1β* mRNA expression and reduced NLRP3, ASC, pro-caspase-1, cleaved caspase-1, GSDMD-F, and GSDMD-N protein levels in LPS-challenged mice. Additional dose-ranging experiments showed partial and component-specific suppression of inflammasome-related proteins at lower Selpercatinib doses, with significant reductions in GSDMD-N at 1 mg/kg and GSDMD-F at 5 mg/kg. Furthermore, the inhibitory effects of 10 mg/kg Selpercatinib on several inflammasome-related proteins were also observed in female mice, supporting the reproducibility of these responses in both sexes, although direct assessment of sex-dependent differences will require further investigation. Reduced NLRP3 inflammasome-related protein levels in the hippocampus are particularly important because these proteins reflect inflammasome activation and downstream neuroinflammatory signaling (22, 37, 52). Because NF-κB-mediated priming is essential for inflammasome activation, and AKT and ERK pathways have also been implicated in NLRP3 inflammasome signaling (42, 53), Selpercatinib-mediated modulation of RET-associated AKT/ERK and NF-κB/CREB signaling may contribute to the suppression of inflammasome-associated neuroinflammatory responses. Thus, the concordance between BV2 microglial data and hippocampal tissue analysis supports the possibility that Selpercatinib limits LPS-induced neuroinflammation through the RET-related NLRP3 inflammasome pathway *in vitro* and *in vivo.* However, an important consideration is the timing of Selpercatinib administration. In the present study, Selpercatinib was administered 30 min after LPS stimulation, before full NLRP3 inflammasome activation would be expected to occur. Thus, our findings indicate that early post-LPS treatment with Selpercatinib attenuates the development of subsequent inflammasome-associated inflammatory responses rather than reversing an already established inflammasome response. Further studies using delayed treatment paradigms will be required to determine whether Selpercatinib can suppress established neuroinflammation and inflammasome activation.

Some limitations of the present study should be considered. The 5 μM concentration of Selpercatinib used *in vitro* is comparable to the reported mean steady-state plasma Cmax of approximately 2.98 μg/mL (approximately 5.67 μM) achieved in patients receiving Selpercatinib at 160 mg twice daily (54). However, plasma exposure cannot be directly extrapolated to brain exposure, and the clinically achievable free concentration of Selpercatinib in the human brain remains unclear despite its reported intracranial efficacy (5). Previous pharmacokinetic studies have demonstrated measurable brain exposure to Selpercatinib in mice following oral administration at 10 mg/kg (55, 56); however, brain or hippocampal drug concentrations were not directly measured after the 10 mg/kg intraperitoneal dose administered in the present study. In addition, although genetic RET knockdown significantly reproduced the anti-inflammatory effects of Selpercatinib, a second structurally unrelated RET inhibitor was not examined; therefore, additional RET-independent pharmacological effects of Selpercatinib cannot be completely excluded. Also, BV2 cells are an immortalized murine microglial cell line and may not fully recapitulate the molecular and functional characteristics of primary microglia or the complex CNS microenvironment. Further studies assessing brain pharmacokinetics, employing additional RET-selective approaches, and validating the findings in primary mouse or human iPSC-derived microglia will be required to establish their broader physiological and translational relevance. Despite the additional dose-ranging and female-mouse validation experiments, the present *in vivo* study was limited to acute endpoints of up to 24.5 h and did not include behavioral or neurological functional assessments, warranting further studies using longer-term models and functional outcomes.

In summary, this study demonstrates that early post-LPS treatment with Selpercatinib attenuates the development of microglia-mediated inflammatory responses through modulation of RET-associated signaling and NLRP3 inflammasome activation. In LPS-stimulated BV2 microglia, Selpercatinib suppressed RET phosphorylation, attenuated AKT/ERK and NF-κB/CREB signaling, modulated microglial activation marker expression, and reduced inflammasome-related protein expression. These anti-inflammatory effects were supported in LPS-challenged mice by reduced hippocampal proinflammatory cytokine expression and inflammasome activation. Collectively, these findings provide a mechanistic basis for further investigating Selpercatinib as an early intervention agent for neuroinflammatory responses, while additional studies using delayed treatment paradigms are required to determine its efficacy against established neuroinflammation.

## Data Availability

The original contributions presented in the study are included in the article/**Supplementary Material**. Further inquiries can be directed to the corresponding authors.

## Glossary

AKT: protein kinase B
ANOVA: analysis of variance
ASC: apoptosis-associated speck-like protein containing a CARD
BCA: bicinchoninic acid
CCK-8: Cell Counting Kit-8
CNS: central nervous system
CO₂: carbon dioxide
COX-2: cyclooxygenase-2
CREB: cAMP response element-binding protein
DAM: disease-associated microglia
DAPI: 4′,6-diamidino-2-phenylindole
DMEM: Dulbecco’s modified Eagle medium
DMSO: dimethyl sulfoxide
ERK: extracellular signal-regulated kinase
FBS: fetal bovine serum
GAPDH: glyceraldehyde-3-phosphate dehydrogenase
GDNF: glial cell line-derived neurotrophic factor
GSDMD: gasdermin D
GSDMD-F: full-length gasdermin D
GSDMD-N: N-terminal gasdermin D
HEPES: 4-(2-hydroxyethyl)-1-piperazineethanesulfonic acid
HRP: horseradish peroxidase
IACUC: Institutional Animal Care and Use Committee
iNOS: inducible nitric oxide synthase
i.p.: intraperitoneal
IL: interleukin
LPS: lipopolysaccharide
MGnD: microglial neurodegenerative phenotype
mRNA: messenger RNA
NF-κB: nuclear factor-κB
NLRP3: NOD-like receptor family pyrin domain-containing 3
PBS: phosphate-buffered saline
PCR: polymerase chain reaction
PEG300: polyethylene glycol 300
PI3K: phosphoinositide 3-kinase
PVDF: polyvinylidene difluoride
qPCR: quantitative real-time polymerase chain reaction
RAS: rat sarcoma virus
RET: rearranged during transfection
RIPA: radioimmunoprecipitation assay
RRID: Research Resource Identifier
SD: standard deviation
SDS-PAGE: sodium dodecyl sulfate-polyacrylamide gel electrophoresis
TLR4: Toll-like receptor 4
TNF: tumor necrosis factor
