# Supplementary material for: Selpercatinib mitigates microglia-mediated neuroinflammation by inhibiting RET signaling and NLRP3 inflammasome activation
Source: Front Immunol. 2026 Sep 23;17:1899666. doi: 10.3389/fimmu.2026.1899666 (PMC13642775; doi:10.3389/fimmu.2026.1899666)
Supplement: Supplementary file 1 [file DataSheet1.docx]

Supplementary Material

Selpercatinib mitigates microglia-mediated neuroinflammation by inhibiting RET signaling and NLRP3 inflammasome activation

Jung Ki Min^1†^, Daeun Park^2†^, Hanwoong Woo^1†^, Sohee Kim^2^, Juyeon Lee^2^, Yeonsu Kim^2^, Jeong-im Choi^2^, Eunbi Lim^1^, Sehyun Chae^1,3^* and Jieun Kim^1,2,4^*

^1^Multidimensional Genomics Research Center, Kangwon National University, Chuncheon, Republic of Korea,

^2^Department of Bio-Health Convergence, Kangwon National University, Chuncheon, Republic of Korea

^3^Division of Chemical Engineering and Bioengineering, College of Art Culture and Engineering, Kangwon National University, Chuncheon, Republic of Korea,

^4^Department of Bio-Health Technology, College of Biomedical Science, Kangwon National University, Chuncheon, Republic of Korea,

† These authors contributed equally to this work.

*** Correspondence:**

Sehyun Chae. Ph.D.
[shchae@kangwon.ac.kr](mailto:shchae@kangwon.ac.kr)

Jieun Kim. Ph.D.

[jieunkim@kangwon.ac.kr](mailto:jieunkim@kangwon.ac.kr)

**Supplementary Figure 1. Effects of LPS stimulation on GDNF secretion and RET phosphorylation in BV2 microglia. (A)** BV2 microglial cell viability following 24 h co-treatment with LPS (200 ng/mL) and/or Selpercatinib (5 μM), assessed by the CCK-8 assay (n=9/group). No significant differences in cell viability were observed among the treatment groups. **(B)** Western blot analysis of secreted GDNF following LPS stimulation for 45 min, 6 h, or 24 h. Secreted GDNF was detectable under basal conditions, and LPS stimulation did not significantly alter GDNF levels at any of the examined time points (n=3/group). **(C)** Western blot analysis of p-RET (Tyr1062) and total RET levels following LPS stimulation for 45 min, 6 h, or 24 h. Both total RET and p-RET (Tyr1062) were detectable under basal conditions. LPS stimulation for 45 min or 6 h did not significantly alter p-RET levels, whereas prolonged LPS stimulation for 24 h significantly reduced p-RET (Tyr1062) levels without altering total RET levels (n=3/group). All values are presented as the mean ± SD. *p < 0.05. The n values indicate independent biological replicates.

**Supplementary Figure 2. Effects of lower doses of Selpercatinib on LPS-induced hippocampal inflammasome-related protein expression in male mice.** **(A)** Schematic of the experimental protocol. Male mice were intraperitoneally injected with LPS (10 mg/kg), followed by Selpercatinib administration at 1 or 5 mg/kg using the same treatment schedule as in the primary *in vivo* experiment. Hippocampal tissues were collected 24.5 h after LPS administration. **(B)** Western blot analysis of NLRP3, ASC, pro-caspase-1, cleaved caspase-1, GSDMD-F, and GSDMD-N. Selpercatinib at 1 mg/kg significantly reduced GSDMD-N levels, whereas Selpercatinib at 5 mg/kg significantly reduced GSDMD-F levels compared with those in the LPS group (n=4/group). All values are presented as the mean ± SD. *p < 0.05, **p < 0.01. The n values indicate independent biological replicates. Cont, control; LPS, LPS-treated group; L+Sel, LPS + Selpercatinib.

**Supplementary Figure 3. Selpercatinib attenuates LPS-induced hippocampal inflammasome-related responses in female mice.** **(A)** Schematic of the experimental protocol. Female mice were intraperitoneally injected with LPS (10 mg/kg), followed by Selpercatinib administration at 10 mg/kg using the same treatment schedule as in the primary *in vivo* experiment. Hippocampal tissues were collected 24.5 h after LPS administration. **(B)** Western blot analysis showed that Selpercatinib significantly reduced NLRP3, ASC, pro-caspase-1, cleaved caspase-1, GSDMD-F, and GSDMD-N levels compared with those in the LPS group in the hippocampus of female mice (n=3-5/group). All values are presented as the mean ± SD. *p < 0.05, **p < 0.01. The n values indicate independent biological replicates. Cont, control; LPS, LPS-treated group; L+Sel, LPS + Selpercatinib.

| **Gene** | **Forward Sequence (5’-3’)** | **Reverse Sequence (5’-3’)** |
| --- | --- | --- |
| ***il-1β*** | TGGACCTTCCAGGATGAGGACA | GTTCATCTCGGAGCCTGTAGTG |
| ***il-6*** | TACCACTTCACAAGTCGGAGGC | CTGCAAGTGCATCATCGTTGTTC |
| ***tnf-α*** | TTCTGTCTACTGAACTTCGGGGTGATCGGTCC | GTATGAGATAGCAAATCGGCTGACGGTGTGGG |
| ***cox-2*** | GCGACATACTCAAGCAGGAGCA | AGTGGTAACCGCTCAGGTGTTG |
| ***inos*** | ATGTCCGAAGCAAACATCAC | TAATGTCCAGGAAGTAGGTG |
| ***ccl-2*** | GCTACAAGAGGATCACCAGCAG | GTCTGGACCCATTCCTTCTTGG |
| ***ccl-7*** | CAGAAGGATCACCAGTAGTCGG | ATAGCCTCCTCGACCCACTTCT |
| ***gapdh*** | CATCACTGCCACCCAGAAGACTG | ATGCCAGTGAGCTTCCCGTTCAG |

**Supplementary Table 1**. List of primers used in real-time PCR for the present study.

**Supplementary Table 2**. List of antibodies used in western blotting and immunostaining for the present study.

| **Antibody** | **Host** | **Dilution**  **(WB/ICC)** | **Cat no.**  **(Company)** |
| --- | --- | --- | --- |
| **anti-p-RET**  **(Tyr1062)** | Rabbit | 1:1000 (WB)  1:200 (ICC) | PA5-104769 (Invitrogen) |
| **anti-RET** | Rabbit | 1:1000 (WB) | A0018 (ABclonal) |
| **anti-p-AKT**  **(Ser473)** | Rabbit | 1:1000 (WB)  1:200 (ICC) | 9271 (Cell Signaling Technology) |
| **anti-AKT** | Rabbit | 1:1000 (WB) | 9272 (Cell Signaling Technology) |
| **anti-p-ERK**  **(Thr202/Tyr204)** | Rabbit | 1:1000 (WB)  1:200 (ICC) | 4377 (Cell Signaling Technology) |
| **anti-ERK** | Rabbit | 1:1000 (WB) | 4695 (Cell Signaling Technology) |
| **anti-p-NF-κB**  **(Ser536)** | Rabbit | 1:1000 (WB)  1:200 (ICC) | 3033 (Cell Signaling Technology) |
| **anti-NF-κB** | Rabbit | 1:1000 (WB) | 8242 (Cell Signaling Technology) |
| **anti-p-CREB**  **(Ser133)** | Rabbit | 1:2000 (WB)  1:200 (ICC) | 9198 (Cell Signaling Technology) |
| **anti-CREB** | Rabbit | 1:1000 (WB) | 9197 (Cell Signaling Technology) |
| **anti-CD16/32** | Rat | 1:200 (ICC) | 80366 (Cell Signaling Technology) |
| **anti-CD206** | Rabbit | 1:200 (ICC) | 24595 (Cell Signaling Technology) |
| **anti-Clec7a** | Rat | 1:200 (ICC) | mabg-mdect-2 (InvivoGen) |
| **anti-Galectin3** | Goat | 1:200 (ICC) | AF1197 (R&D System) |
| **anti-NLRP3** | Rabbit | 1:1000 (In vitro WB)  1:200 (ICC)  1:200 (In vivo WB) | NBP2-12446 (Novus Biologicals) |
| **anti-ASC** | Mouse | 1:50 (In vivo WB) | sc-514414 (Santa Cruz Biotechnology) |
| **anti-Caspase1** | Rabbit | 1:1000 (In vitro WB)  1:100 (ICC)  1:1000 (In vivo WB) | A16792 (ABclonal) |
| **anti-GSDMD** | Rabbit | 1:1000 (In vitro WB)  1:150 (In vivo WB) | NBP2-33422 (Novus Biologicals) |
| **Anti-GDNF** | Rabbit | 1:1000 (WB) | A14639 (ABclonal) |
| **anti-GAPDH** | Rabbit | 1:20000 (In vitro WB)  1:10000 (In vivo WB) | 2118 (Cell Signaling Technology) |
| **anti-CD11b** | Rat | 1:200 (ICC) | 46512 (Cell Signaling Technology) |
| **anti-Iba1** | Mouse | 1:200 (ICC) | sc-32725 (Santa Cruz Biotechnology) |
| **anti-H3** | Rabbit | 1:10000 (WB) | 9715 (Cell Signaling Technology) |

**Supplementary Table 3**. Statistical p-values are presented in this study.

| **Figure** | | **Comparison** | | **Significance** | | **p-value** |
| --- | --- | --- | --- | --- | --- | --- |
| 1B | | 0 μM vs 10 μM | | **** | | <0.0001 |
| 1C  (*il-1β / gapdh*) | | Cont vs LPS | | **** | | <0.0001 |
|  |  | LPS vs L+Sel | | **** | | <0.0001 |
| 1C  (*il-6 / gapdh*) | | Cont vs LPS | | **** | | <0.0001 |
|  |  | LPS vs L+Sel | | **** | | <0.0001 |
| 1C  (*tnf-α / gapdh*) | | Cont vs LPS | | **** | | <0.0001 |
|  |  | LPS vs L+Sel | | **** | | <0.0001 |
| 1C  (*cox-2 / gapdh*) | | Cont vs LPS | | **** | | <0.0001 |
|  |  | LPS vs L+Sel | | **** | | <0.0001 |
| 1C  (*inos / gapdh*) | | Cont vs LPS | | ** | | 0.0030 |
|  |  | LPS vs L+Sel | | * | | 0.0216 |
| 1C  (*ccl-2 / gapdh*) | | Cont vs LPS | | **** | | <0.0001 |
|  |  | LPS vs L+Sel | | * | | 0.0124 |
| 1C  (*ccl-7 / gapdh*) | | Cont vs LPS | | **** | | <0.0001 |
|  |  | LPS vs L+Sel | | * | | 0.0237 |
| 1D | | LPS vs L+Sel | | **** | | <0.0001 |
| 1E | | LPS vs L+Sel | | * | | 0.0349 |
| 1F  (p-RET / GAPDH) | | LPS vs L+Sel | | * | | 0.0263 |
|  |  | siSCR+L vs siRET+L | | ** | | 0.0065 |
| 1F  (RET / GAPDH) | | siSCR+L vs siRET+L | | ** | | 0.0094 |
| 1G  (*il-1β / gapdh*) | | Cont vs LPS | | **** | | <0.0001 |
|  |  | LPS vs L+Sel | | **** | | <0.0001 |
|  |  | siSCR+L vs siRET+L | | **** | | <0.0001 |
| 1G  (*il-6 / gapdh*) | | Cont vs LPS | | *** | | 0.0001 |
|  |  | LPS vs L+Sel | | *** | | 0.0007 |
|  |  | siSCR+L vs siRET+L | | **** | | <0.0001 |
| 1G  (*tnf-α / gapdh*) | | Cont vs LPS | | **** | | <0.0001 |
|  |  | LPS vs L+Sel | | ** | | 0.0027 |
|  |  | siSCR+L vs siRET+L | | **** | | <0.0001 |
| 1G  (*cox-2 / gapdh*) | | Cont vs LPS | | **** | | <0.0001 |
|  |  | LPS vs L+Sel | | **** | | <0.0001 |
|  |  | siSCR+L vs siRET+L | | **** | | <0.0001 |
| 2A | | Cont vs LPS | | ** | | 0.0015 |
|  |  | LPS vs L+Sel | | *** | | 0.0003 |
| 2B | | Cont vs LPS | | **** | | <0.0001 |
|  |  | LPS vs L+Sel | | **** | | <0.0001 |
| 2C | | Cont vs LPS | | ** | | 0.0058 |
|  |  | LPS vs L+Sel | | *** | | 0.0002 |
| 2D | | Cont vs LPS | | ** | | 0.0024 |
|  |  | LPS vs L+Sel | | * | | 0.0175 |
| 2E  (p-AKT / GAPDH) | | Cont vs LPS | | *** | | 0.0003 |
|  |  | LPS vs L+Sel | | **** | | <0.0001 |
|  |  | siSCR+L vs siRET+L | | **** | | <0.0001 |
| 2E  (p-ERK / GAPDH) | | Cont vs LPS | | **** | | <0.0001 |
|  |  | LPS vs L+Sel | | **** | | <0.0001 |
|  |  | siSCR+L vs siRET+L | | **** | | <0.0001 |
| 3A | | Cont vs LPS | | *** | | 0.0001 |
|  |  | LPS vs L+Sel | | * | | 0.0255 |
| 3B | | Cont vs LPS | | ** | | 0.0024 |
|  |  | LPS vs L+Sel | | * | | 0.0156 |
| 3C | | Cont vs LPS | | * | | 0.0222 |
|  |  | LPS vs L+Sel | | *** | | 0.0004 |
| 3D | | Cont vs LPS | | *** | | 0.0001 |
|  |  | LPS vs L+Sel | | *** | | 0.0002 |
| 3E | | Cont vs LPS | | * | | 0.0444 |
|  |  | LPS vs L+Sel | | * | | 0.0414 |
|  |  | siSCR+L vs siRET+L | | ** | | 0.0072 |
| 3F | | Cont vs LPS | | ** | | 0.0020 |
|  |  | LPS vs L+Sel | | ** | | 0.0038 |
|  |  | siSCR+L vs siRET+L | | * | | 0.0176 |
| 4B | | Cont vs LPS | | **** | | <0.0001 |
|  |  | LPS vs L+Sel | | **** | | <0.0001 |
| 4C | | LPS vs L+Sel | | ** | | 0.0018 |
| 4E | | Cont vs LPS | | ** | | 0.0013 |
|  |  | LPS vs L+Sel | | * | | 0.0241 |
| 4F | | Cont vs LPS | | *** | | 0.0002 |
|  |  | LPS vs L+Sel | | ** | | 0.0076 |
| 4G | | Cont vs LPS | | **** | | <0.0001 |
|  |  | LPS vs L+Sel | | ** | | 0.0017 |
| 5B  (NLRP3 / GAPDH) | | Cont vs LPS | | **** | | <0.0001 |
|  |  | LPS vs L+Sel | | * | | 0.0150 |
| 5B  (ASC / GAPDH) | | Cont vs LPS | | ** | | 0.0026 |
|  |  | LPS vs L+Sel | | ** | | 0.0069 |
| 5B  (Pro-casp1 / GAPDH) | | Cont vs LPS | | **** | | <0.0001 |
|  |  | LPS vs L+Sel | | ** | | 0.0045 |
| 5B  (Cleaved-casp1 / GAPDH) | | Cont vs LPS | | ** | | 0.0095 |
|  |  | LPS vs L+Sel | | * | | 0.0246 |
| 5B  (GSDMD-F / GAPDH) | | Cont vs LPS | | *** | | 0.0008 |
|  |  | LPS vs L+Sel | | * | | 0.0136 |
| 5C | | Cont vs LPS | | **** | | <0.0001 |
|  |  | LPS vs L+Sel | | **** | | <0.0001 |
| 5E | | Cont vs LPS | | *** | | 0.0002 |
|  |  | LPS vs L+Sel | | *** | | 0.0004 |
| 5F | | Cont vs LPS | | ** | | 0.0019 |
|  |  | LPS vs L+Sel | | ** | | 0.0015 |
| 6B | | Cont vs LPS | | *** | | 0.0001 |
|  |  | LPS vs L+Sel | | * | | 0.0120 |
| 6D  (NLRP3 / GAPDH) | | Cont vs LPS | | * | | 0.0184 |
|  |  | LPS vs L+Sel | | ** | | 0.0051 |
| 6D  (ASC / GAPDH) | | LPS vs L+Sel | | * | | 0.0339 |
| 6D  (Pro-casp1 / GAPDH) | | Cont vs LPS | | ** | | 0.0070 |
|  |  | LPS vs L+Sel | | * | | 0.0241 |
| 6D  (Cleaved-casp1 / GAPDH) | | Cont vs LPS | | * | | 0.0189 |
|  |  | LPS vs L+Sel | | * | | 0.0103 |
| 6D  (GSDMD-F / GAPDH) | | Cont vs LPS | | * | | 0.0494 |
|  |  | LPS vs L+Sel | | * | | 0.0110 |
| 6D  (GSDMD-N / GAPDH) | | Cont vs LPS | | ** | | 0.0049 |
|  |  | LPS vs L+Sel | | ** | | 0.0045 |
| **Supplementary Figure** | **Comparison** | | **Significance** | | **p-value** | |
| 1C | | LPS 0 h vs LPS 24 h | | * | | 0.0181 |
| 2B  (ASC / GAPDH) | | Cont vs LPS | | * | | 0.0456 |
| 2B  (Pro-casp1 / GAPDH) | | Cont vs LPS | | ** | | 0.0086 |
| 2B  (GSDMD-F / GAPDH) | | Cont vs LPS | | * | | 0.0493 |
|  |  | LPS vs L+Sel 5 mg/kg | | * | | 0.0252 |
| 2B  (GSDMD-N / GAPDH) | | Cont vs LPS | | * | | 0.0452 |
|  |  | LPS vs L+Sel 1 mg/kg | | * | | 0.0491 |
| 3B  (NLRP3 / GAPDH) | | Cont vs LPS | | * | | 0.0489 |
|  |  | LPS vs L+Sel | | * | | 0.0302 |
| 3B  (ASC / GAPDH) | | LPS vs L+Sel | | * | | 0.0400 |
| 3B  (Pro-casp1 / GAPDH) | | Cont vs LPS | | ** | | 0.0078 |
|  |  | LPS vs L+Sel | | * | | 0.0139 |
| 3B  (Cleaved-casp1 / GAPDH) | | Cont vs LPS | | * | | 0.0160 |
|  |  | LPS vs L+Sel | | ** | | 0.0070 |
| 3B  (GSDMD-F / GAPDH) | | Cont vs LPS | | * | | 0.0149 |
|  |  | LPS vs L+Sel | | * | | 0.0176 |
| 3B  (GSDMD-N / GAPDH) | | Cont vs LPS | | * | | 0.0240 |
|  |  | LPS vs L+Sel | | * | | 0.0131 |
